# The Indispensable Role of Histone Methyltransferase *Po*Dot1 in Extracellular Glycoside Hydrolase Biosynthesis of *Penicillium oxalicum*

**DOI:** 10.3389/fmicb.2019.02566

**Published:** 2019-11-07

**Authors:** Yanan Li, Yueyan Hu, Kaili Zhao, Yunjun Pan, Yinbo Qu, Jian Zhao, Yuqi Qin

**Affiliations:** ^1^National Glycoengineering Research Center and State Key Laboratory of Microbial Technology, Shandong University, Qingdao, China; ^2^College of Life Sciences, Henan Agricultural University, Zhengzhou, China; ^3^Shandong Provincial Key Laboratory of Carbohydrate Chemistry and Glycobiology, Shandong University, Qingdao, China

**Keywords:** *Penicillium oxalicum*, histone methyltransferase, Dot1, glycoside hydrolases, regulation

## Abstract

Histone methylation is associated with transcription regulation, but its role for glycoside hydrolase (GH) biosynthesis is still poorly understood. We identified the histone H3 lysine 79 (H3K79)-specific methyltransferase *Po*Dot1 in *Penicillium oxalicum*. *Po*Dot1 affects conidiation by regulating the transcription of key regulators (BrlA, FlbC, and StuA) of asexual development and is required in normal hyphae septum and branch formation by regulating the transcription of five septin-encoding genes, namely, *aspA*, *aspB*, *aspC*, *aspD*, and *aspE*. Tandem affinity purification/mass spectrometry showed that *Po*Dot1 has no direct interaction with transcription machinery, but it affects the expressions of extracellular GH genes extensively. The expression of genes (*amy15A*, *amy13A*, *cel7A/cbh1*, *cel61A*, *chi18A*, *cel3A/bgl1*, *xyn10A*, *cel7B/eg1*, *cel5B/eg2*, and *cel6A/cbh2*) that encode the top 10 GHs was remarkably downregulated by *Podot1* deletion (Δ*Podot1*). Consistent with the decrease in gene transcription level, the activities of amylases and cellulases were significantly decreased in Δ*Podot1* mutants in agar (solid) and fermentation (liquid) media. The repression of GH gene expressions caused by *Po*Dot1 deletion was not mediated by key transcription factors, such as AmyR, ClrB, CreA, and XlnR, but was accompanied by defects in global demethylated H3K79 (H3K79me2) and trimethylated H3K79 (H3K79me3). The impairment of H3K79me2 on specific GH gene loci was observed due to *Po*Dot1 deletion. The results implies that defects of H3K79 methylation is the key reason of the downregulated transcription level of GH-encoding genes and reveals the indispensable role of PoDot1 in extracellular GH biosynthesis.

## Introduction

*Penicillium* is a well-known fungi that plays important roles in biotechnology and in the medical and food industries (Arora, 2004). *P. oxalicum* has a high representation of extracellular proteins that are involved in plant cell wall degradation (Qu et al., 1991; Fang et al., 2010) and produces various glycoside hydrolases (GHs), including typical cellulases, hemicellulases, amylases, other carbohydrate-active enzymes, and cellulolytic enzyme-related regulators (Liu et al., 2013). These GHs and regulators are conserved in many other cellulolytic enzyme-producing filamentous fungi, such as *Trichoderma reesei* (Martinez et al., 2008), *Aspergillus* spp. (Florencio et al., 2016), and *Neurospora crassa* (Tian et al., 2009). *P. oxalicum* is a good system that can be used to elucidate the regulatory mechanism of gene expression of GHs.

Glycoside hydrolase production is tightly controlled at the transcriptional level (Lynd et al., 2008). Several conserved positive or negative transcriptional factors (TFs) have been characterized as key regulators, such as positive regulators encoded by *xyr1*/*xlnR* and *clr-2*/*clrB* (Mach-Aigner et al., 2008; Coradetti et al., 2012) and negative regulators encoded by *cre1*/*creA* and *amyR* (Li et al., 2015). In eukaryotes, TFs rarely activate or repress transcription through direct interaction with RNA polymerase. In many cases, they recruit nucleosome modifiers that alter chromatin in the vicinity of a promoter and help or interfere transcription initiation. Recently, reports revealed that the reorganization of local chromatin or modification of histones is involved in the regulation of cellulolytic enzyme gene expression (Mello-de-Sousa et al., 2015, 2016; Cao et al., 2019; Li et al., 2019). For example, substrate-induced transcriptional activation of the MoCel7C cellulase gene in the rice blast fungus *Magnaporthe oryzae* is associated with histone methylation H3 at lysine 4 (H3K4) (Vu et al., 2013).

Gene transcription is critically influenced by chromatin structure and modification status of histone tails in eukaryotic cells (Strauss and Reyes-Dominguez, 2011). Histone lysine methylation, an important epigenetic modification, plays an essential role in the recruitment of chromatin remodeling complexes or subunit of transcriptional machinery (Strauss and Reyes-Dominguez, 2011; Croken et al., 2012) and activates or represses transcription. In general, methylations of lysine 9 (K9) and 27 (K27) on histone H3 and lysine 20 (K20) on histone H4 are correlated with repressed transcription, whereas methylations of lysine 4 (K4), 36 (K36), and 79 (K79) on histone H3 are associated with active transcription (Yao and Cohen, 2002; Peters et al., 2003; Zhang et al., 2009; Gu et al., 2010). These histone lysine methylations are catalyzed by a group of histone lysine-specific methyltransferases (HKMTs), which are usually divided into two classes based on their catalytic domains (Nguyen and Zhang, 2011). One class contains the evolutionarily conserved Su(var)3-9, Enhancer-of-zeste, and Trithorax (SET) domains, such as Set1 and Set2 that perform H3K4 and H3K36 methylations, respectively (Binda, 2013). The other class does not contain the SET domain and consists of only an evolutionarily conserved protein named disruptor of telomeric silencing 1 (Dot1) and its homologs, which perform H3K79 methylation.

Dot1 was initially identified in *Saccharomyces cerevisiae*, and its deletion or overexpression confers defects in telomeric silencing (van Leeuwen et al., 2002; Takahashi et al., 2011; Farooq et al., 2016). Although studies on Dot1 and H3K79 methylation in filamentous fungi are rare, one report indicated the function of Dot1 in producing secondary metabolites aflatoxin in *Aspergillus flavus* (Liang et al., 2017). The result is understandable, because many secondary metabolic gene clusters, including the aflatoxin gene cluster, are near telomeres (Keller et al., 2005; Palmer and Keller, 2010). The putative protein methyltransferase LaeA is also a key regulator of secondary metabolism observed in many fungi. Its ortholog LAE1 controls cellulase gene expression in *T. reesei* (Seiboth et al., 2012). The indispensable role of LaeA in the production of extracellular cellulolytic enzymes and secondary metabolites in *P. oxalicum* was determined (Li et al., 2016; Zhang et al., 2016b). As the distribution of genes encoding plant cell wall-degrading enzymes in the *P. oxalicum* genome often occurred in clusters and was accompanied by secondary metabolic gene clusters near the telomeres of chromosomes (Liu et al., 2013; Zhang et al., 2016b), we aimed to determine whether cellulolytic enzymes in *P. oxalicum* were regulated by Dot1.

We identified the histone methyltransferase *Po*Dot1 in *P. oxalicum*. Characterization of the *Po*Dot1 deletion mutant showed its critical roles in normal fungal development and in the transcription regulation of multiple genes, especially in the regulation of extracellular GH production in *P. oxalicum*.

## Materials and Methods

### Fungal Strains and Culture Conditions

The *P. oxalicum* wild-type (WT) strain 114-2 (CGMCC 5302) was used as a progenitor for transformation experiments. The WT strain and transformants generated in this study were grown at 30°C on agar with 10% wheat bran juice for conidiation, or on Vogel’s minimal medium (50× Vogel’s salt: 125.0 g Na_3_Citrate.2H_2_O, 250.0 g KH_2_PO_4_, 100.0 g NH_4_NO_3_, 10.0 g MgSO_4_.7H_2_O, 5.0 g CaCl_2_.2H_2_O, 0.25 mg biotin, 0.25 g citric acid, 0.25 g ZnSO_4_.7H_2_O, 0.05 g Fe(NH_4_)_2_(SO_4_)_2_.6H_2_O, 12.5 mg CuSO_4_.5H_2_O, 2.5 mg MnSO_4_.H_2_O, 2.5 mg H_3_BO_3_, 2.5 mg Na_2_MoO_4_.2H_2_O, and 1 L of water) plus different carbon sources (2% glucose or 0.5% cellulose or 2% starch) for mycelial growth (Vogel, 1956).

### Phylogenetic Analysis and Domain Architecture Analysis

The amino acid sequences of Dot1 homologous proteins from different species were obtained from the NCBI^1^ database. The softwares Clustal X (Larkin et al., 2007) and MEGA 7.0 (Kumar et al., 2016) were used to construct multiple sequence alignments and construct phylogenetic trees with the neighbor-joining method. The SMART^2^ database (Schultz et al., 1998) and the Pfam^3^ database (Finn et al., 2016) were used for domain analysis of proteins. The domain architecture patterns were constructed in proportion to the corresponding protein sequences.

### Generation of *Po*Dot1-Related Mutants

The genome of the WT strain was used as the template to amplify 1718 base pair (bp) of upstream fragments and 1736 bp of downstream fragments of *Podot1* coding region, respectively. The plasmid pSilent 1 (Nakayashiki et al., 2005) was used as the template to amplify 1890 bp of selective marker gene *hygromycin B* (*hph*). The *Podot1* knockout cassette (4677 bp) was obtained by fusing the upstream fragments, *hph*, and the downstream fragments using double-joint PCR method (Yu et al., 2004). Then the cassette was introduced into the WT strain using PEG-mediated protoplast method developed by Li et al. (2010) to obtain the *Podot1* deletion strain (Δ*Podot1*).

The expression sequence (4219 bp) of *Podot1* including its native promoter (1646 bp), coding sequence (CDS) (1567 bp), and transcriptional terminator (1006 bp) was amplified from the genome of the WT. The marker gene *pyrithiamine hydrobromide* (*ptrA*) (2008 bp) was amplified from plasmid pME2892 (Kubodera et al., 2002). The *Podot1* recovery cassette (6134 bp) was obtained by fusing the marker gene *ptrA* and the expression sequence of *Podot1*. Then, the recovery cassette was introduced into the Δ*Podot1* to obtain the recovery strain (Re*Podot1*).

The *glyceraldehyde-3-phosphate dehydrogenase* (*gpdA*) promoter (1314 bp) was amplified from plasmid pAN7-1 (Itoh and Scott, 1997). The CDS plus terminator (2857 bp) of *Podot1* were amplified from the genome of the WT strain. The marker gene *hph* (1890 bp) was amplified from plasmid pSilent 1. The *Podot1* overexpression cassette (5824 bp) was obtained by fusing promoter *gpdA*, CDS plus terminator of *Podot1*, and marker gene *hph*. Then, the overexpression cassette was introduced into the WT strain to obtain the overexpression strain (OE*Podot1*).

For subcellular localization observation of *Po*Dot1, the *Podot1*-*GFP* fusion cassette was constructed, which including the promoter region and CDS of *Podot1* (1698 bp), the CDS of *GFP* (720 bp), the marker gene *pyrG* (1398 bp), and the terminator of *Podot1* (1535 bp). Then the cassette was introduced into the uracil auxotrophic *P. oxalicum* strain M12 (Qin et al., 2013b) to replace native *Po*Dot1 and obtain *Podot1*-*GFP* fusion strain (*Po*Dot1-GFP).

For tandem affinity purification of *Po*Dot1, the *Podot1*-*HA-FLAG* fusion cassette was constructed, which including the promoter region and CDS of *Podot1*, the CDS of *HA-FLAG* tags, the marker gene *hph*, and the terminator of *Podot1*. Then the cassette was introduced into the WT strain to replace native *Po*Dot1 and obtain *Podot1*-*HA-FLAG*-labeled strain (*Po*Dot1-TAP).

All mutants were verified by diagnostic PCR and Southern blot. For Southern blot, the genomes of the transformants were fully digested by applicable restriction enzymes. The DNA fragments were separated by 0.75% agarose gel electrophoresis, and were transferred to a Hybond-N^+^ nylon membrane (GE Healthcare, United States). The desired DNA probe was amplified and subjected to Southern blot according to the instructions of DIG-High Prime DNA labeling and detection starter kit (Roche, Switzerland). The primers used for PCR amplification were shown in Supplementary Table S1. The strategies and results of Southern blot were shown in Supplementary Image S1.

### Phenotypic Analysis of *P. oxalicum* Strains

For observation of colony morphology, 1 μL of fresh spore suspension (1 × 10^7^ spores/mL) was spotted on potato dextrose agar (PDA) or Vogel’s agar with 2% glucose as carbon source, and then, was cultivated at 30°C for 5 days. The colony diameters were measured. For spore count, 120 μL of fresh spore suspension (1 × 10^7^ spores/mL) was spread evenly onto glucose agar plates. After cultivated at 30°C for 5 days, 5-mm diameter agars were taken from each plate, and spores on which were washed with 2 mL of physiological saline by vortexing. Then the spores were counted using a blood cell counting plate. For microscopic observation of hyphae and conidiation, the mycelial samples were stained with lactophenol cotton blue reagent (0.05 g cotton blue, 20.0 g phenol crystals, 40.0 mL glycerol, 20.0 mL lactic acid, and 20.0 mL distilled water) for 5 h. The mycelium and conidia at different growth stages were observed under a Nikon phase contrast microscope. For observation of septa and hyphal branching, 50 hyphal elements were randomly selected and observed using the Nikon Eclipse 80i microscope with a 40× objective. The distance between the septa was measured from digitalized photographs at 400× magnification using NIS-Elements AR 3.1 software. Hyphal growth unit length (*L*_*hgu*_) was calculated according to method described by Wiebe and Trinci (1991). The biomass under the condition of glucose carbon source was determined by the dry weight method (Yao et al., 2015). The biomass under the condition of cellulose carbon source was reflected by the intracellular protein content (Zhang et al., 2016a). Three replicates were included for each strain sample.

### Subcellular Localization Observation

Hyphae of *Po*Dot1-GFP strain were observed under a high sensitivity laser scanning confocal microscope (ZEISS LSM780) (Carl Zeiss, Germany). The nuclei were stained in the dark for 15 min by Hoechest 33342 (Sigma, United States). The green fluorescence of *Po*Dot1-GFP was observed by excitation light at 488 nm, and the blue nuclei stained with Hoechest 33342 were observed with excitation light at 405 nm. The subcellular localization of *Po*Dot1 was determined by comparing whether green fluorescence and blue fluorescence overlap.

### Real-Time Quantitative PCR

Fresh spores (1 × 10^7^ spores/mL) were precultured in Vogel’s medium with 2% glucose at 30°C for 22 h. Equal mycelia was collected through vacuum filtration and was then transferred to fermentation medium (0.3 g/50 mL) with 2% glucose or with 1% cellulose plus 1% wheat bran as carbon sources. Fresh mycelia after 24 h of cultivation (30°C, 200 rpm) in glucose or cellulose medium were harvested and fully ground in liquid nitrogen. Total RNA was extracted with RNAiso Plus reagent (Takara, Japan). Genomic DNA removal and cDNA synthesis were performed using PrimeScript^TM^ RT reagent Kit With gDNA Eraser (Takara, Japan). The obtained cDNA was used as the template for quantitative PCR using LightCycler^®^ 480 (Roche, United States) with SYBR Premix Ex Taq^TM^ (Takara, Japan). The primers used were listed in Supplementary Table S1. For each sample, three replicates were conducted. Gene expression copy numbers were calculated using the standard curves constructed for each gene, and the data were then normalized with the expression levels of the *actin* gene.

### Gene Expression Profiling and Data Analysis

Strains were cultivated as previous described in the section “Real-Time Quantitative PCR.” Fresh mycelia of WT (three biological replicates) and Δ*Podot1* (three biological replicates) were harvested and ground in liquid nitrogen after 24 h of cultivation in glucose or cellulose medium. Total RNA was extracted with RNAiso Plus reagent (Takara, Japan), and was incubated with 10 U DNase I (Takara, Japan) at 37°C for 30 min to remove genomic DNA. The quality of mRNA sample was assayed [OD260/OD280: 1.8–2.2; OD260/OD230: >1.5; RNA integrity number (RIN): >8.0] before subsequent library construction. Expression profiling based on BGISEQ-500 RNA-Seq was performed by the Beijing Genomics Institute (BGI, Shenzhen, China). Saturation analysis of each sample was performed to determine their availability for omics analysis. Sequenced reads were mapped against reference genome using HISAT (Kim et al., 2015) and mapped against predicted transcripts using Bowtie2 (Langmead et al., 2019). The filtered clean reads for each gene were normalized to fragments per kilobase transcriptome per million mapped reads (FPKM) for differential expression analysis. Significantly different expression between samples were identified through a significance test with combined thresholds (fold change ≥ 2, diverge probability ≥ 0.8) (Audic and Claverie, 1997). GO database^4^ and Blast2GO^5^ were used for GO annotation and function enrichment analysis with threshold at FDR ≤ 0.05 (Conesa et al., 2005). Genesis software was used for Cluster analysis (Sturn et al., 2002).

### Extracellular Glycoside Hydrolase Activity Assay

Strains were precultured and transferred to fermentation medium with 1% cellulose plus 1% wheat bran as carbon sources as the methods described in the section “Real-Time Quantitative PCR.” Fermentation supernatants of different strains at different time points were obtained to determine the extracellular GH activity. Five types of enzyme activities were assayed by the methods described by Li et al. (2015) and Yao et al. (2015).

For the assay of amylase activity, 1.5 mL of 1% starch (Sigma–Aldrich, United States) (1 g dissolved in 100 mL HAc-NaAc buffer) was used as substrate. The substrate mixed with 0.5 mL of diluted culture supernatants in a 25 mL colorimetric tube. Then, the mixture was incubated in a 50°C water bath for 10 min. Three milliliters of 3,5-dinitrosalicylic acid (DNS) reagent (10 g DNS, 20 g sodium hydroxide, 200 g sodium potassium tartrate, 2.0 g redistilled phenol, and 0.50 g sodium sulfite anhydrous per 1000 mL DNS reagent) was then added to stop enzymatic reaction. The tubes were placed in boiling water for 10 min. Finally, 20 mL of distilled water was added and mixed, and the absorbance of the reaction mixture was measured at 540 nm to determine enzyme activities. The same DNS method was used for the assay of filter paper activity (FPA) and endoglucanase (CMCase) activity, with 50 ± 1 mg Whatman No. 1 filter paper and 1% carboxymethylcellulose sodium salt (CMC-Na) (Sigma–Aldrich, United States) as substrates, respectively, and with the enzymatic hydrolysis time at 50°C of 60 and 30 min, respectively.

For the assay of cellobiohydrolase (pNPCase) and extracellular β-glucosidase (pNPGase) activity, 1 mg/mL pNPC and 1 mg/mL pNPG (Sigma–Aldrich, United States) were used as substrates, respectively. Briefly, 100 μL of diluted culture supernatants and 50 μL of substrates were mixed and incubated at 50°C for 30 min. 150 μL of 10% Na_2_CO_3_ (w/v) was then added to terminate the reaction, and the absorbance of the reaction mixture was measured at 420 nm to determine the enzyme activities.

Cellulolytic enzyme activities were normalized to the ratio of corresponding biomass. The concentration of intracellular protein was determined to indicate the biomass. The normalized cellulolytic enzyme activities (IU/mg) represented the enzyme activity produced by the mycelium corresponding to 1 mg intracellular protein. One enzyme activity unit was defined as the amount of enzyme required for producing 1 μmol glucose or pNP per minute under the assayed conditions. Three biological triplicates were performed for all enzyme analysis. The mean values and standard deviations were calculated.

### Western Blotting Analysis

Strains were precultured and transferred to fermentation medium with 1% cellulose plus 1% wheat bran as carbon sources as the methods described in the section “Real-Time Quantitative PCR.” Fresh mycelia after 24 h of cultivation were harvested and fully ground in liquid nitrogen for total protein extraction. 0.2 g mycelia was mixed with 0.5 mL extraction buffer [50 mM Tris–HCl (pH 7.5), 150 mM NaCl, 1% NP-40, 1 mM Phenylmethanesulfonyl fluoride (PMSF), 0.1% protease inhibitor cocktail (Sigma, United States)] in an ice bath for 30 min and centrifugated at 12,000 rpm at 4°C for 10 min to collect supernatant. Equal amounts of total protein were separated by SDS polyacrylamide gel electrophoresis (SDS-PAGE) on a 15% gel and were transferred to nitrocellulose membrane (Pall Corp., Ann Arbor, MI, United States) using a Bio-Rad electroblotting apparatus. Anti-H3K79me2 antibody (ab3594, Abcam, United Kingdom) and anti-H3K79me3 antibody (OM256861, OmnimAbs, United States) were employed to detect H3K79 methylation. Anti-histone H3 antibody (OM256785, OmnimAbs, United States) was used as loading control. ECL chemiluminescence solution (freshly made) was used to image the target protein strips. The signal strength of Western blot band was further quantified by the software ImageJ.

### ChIP-qPCR Analysis

Chromatin immunoprecipitation (ChIP) assays were performed as previously described (Reyes-Dominguez et al., 2010; Chujo and Scott, 2014) with modifications. Briefly, the hyphae cultivated in liquid fermentation medium were cross-linked with 1% formaldehyde for 10 min, followed by mixing with 125 mM glycine for 5 min to stop the fixation. Collected hyphae were ground in liquid nitrogen and lysed in lysis buffer (50 mM HEPES pH 7.5, 150 mM NaCl, 1 mM EDTA, 0.5% Triton X-100, 0.1% sodium deoxycholate, 0.1% SDS, 1 mM PMSF, 0.1% protease inhibitor cocktail), and followed by centrifugation to obtain chromatin. The chromatin was broken into fragments of approximately 500 bp by sonication at 35% power output. Immunoprecipitation (IP) was performed with anti-H3K79me2 antibody (ab3594, Abcam, United Kingdom) and protein A/G magnetic beads (Thermo Fisher Scientific, MA, United States) with equal amounts of extracted chromatin (1 mg). The obtained IP products and 0.1 mg input chromatin DNA (without IP) of each sample were subjected to RNase digestion to remove RNA, as well as high heat and proteinase K digestion to reverse crosslinks. Then IP DNA and input DNA were purified using phenol extraction and ethanol precipitation. Quantitative PCR was finally performed by using LightCycler^®^ 480 (Roche, Indianapolis, IN, United States). Primers used here were shown in Supplementary Table S1. The relative enrichment of IP DNA was calculated by the Input% method as follows (where Ct = the number of qPCR cycles required to reach the threshold):

ChIP efficiency = 2^–Δ^ ^*Ct*^ × 100%
ΔCt = Ct_*IP*_ − (Ct_*Input*_ − log_2_10)

Three biological triplicates were performed for all strain samples.

### Statistical Analysis

The statistical significance tests were performed with a one-tailed homoscedastic (equal variance) *t*-test. The mean values, standard deviations, and *P*-values were calculated in all quantitative analysis. *P*-values ≤ 0.05 were considered statistically significant.

### Tandem Affinity Purification–Mass Spectrometry

The experiment of tandem affinity purification–mass spectrometry (TAP–MS) was performed according to the methods of Puig et al. (2001). The WT strain, *Po*Dot1-TAP, and positive control strain were cultured in fermentation medium with 1% cellulose plus 1% wheat bran as carbon sources at 30°C, 200 rpm for 60 h. About 40 g of mycelium for each sample was collected by vacuum filtration and liquid nitrogen grinding. Then, via the tandem affinity purification by Ezview^TM^ Red ANTI-FLAG M2 Affinity resin (Sigma–Aldrich, United States) and ANTI-HA resin (Sigma–Aldrich, United States), the eluent containing *Po*Dot1 and interacted proteins was obtained. The two-step eluent was analyzed by silver staining of SDS-PAGE, Western blot, and mass spectrometry to determine the presence of the bait protein *Po*Dot1 and interacted proteins of *Po*Dot1. The mass spectrometry data were analyzed using the MASCOT engine (Matrix Science, United Kingdom; Version 2.2) for a non-redundant international protein index from the European Bioinformatics Institute. Three biological repeats were set for each strain. Proteins that were unique to *Po*Dot1-TAP eluent and with unique pep count ≥ 3 were selected for further bioinformatics analysis.

### Accession Numbers

The Whole Genome Shotgun projects were deposited in DDBJ/EMBL/GenBank under the accession number AGIH00000000. The raw data of expression profiling were deposited in NCBI’s Gene Expression Omnibus (GEO) database under the accession number GSE136585.

## Results

### Identification of H3K79-Specific Histone Methyltransferase Dot1 in *P. oxalicum*

**FIGURE 1:**
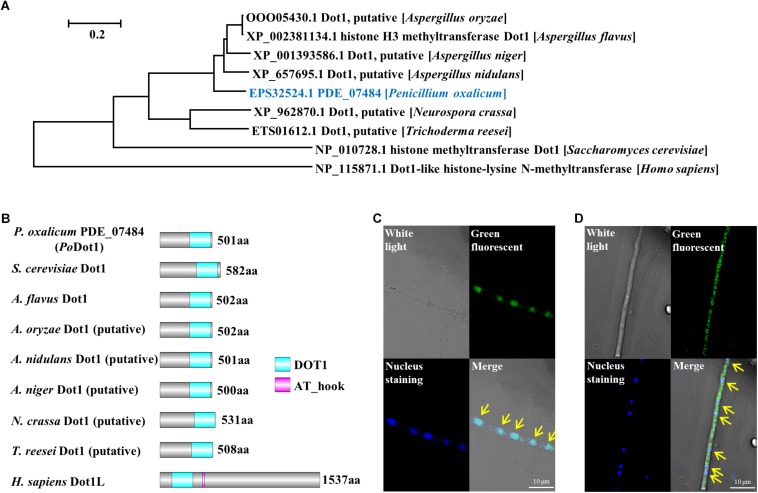
Identification of histone methyltransferase *Po*Dot1 in *P. oxalicum*. **(A)** Phylogenetic analysis of *Po*Dot1 orthologs. **(B)** The domain architecture analysis of *Po*Dot1 orthologs. The maps were constructed with equal proportions of the respective sequences. A diagram of domains of Dot1 orthologs: DOT1 and AT_hook domains. **(C,D)** Subcellular localization of *Po*Dot1. Upper left, white light; upper right, green fluorescence (green dots); bottom left, nuclear staining (blue dots); bottom right, merged. Yellow arrows indicate the location that showed the overlap of green fluorescence and nuclear staining on the merged image.

We performed BLASTp using the sequence of *S. cerevisiae* Dot1p as a query to find Dot1 in *P. oxalicum*. PDE_07484 (GenBank EPS32524.1), a single ortholog, was identified within the *P. oxalicum* genome and named as *Po*Dot1. It is a 501-amino acid protein that shares 37% identity with *S. cerevisiae* Dot1p (van Leeuwen et al., 2002), 73% identity with Dot1 orthologs in *A. flavus* (Liang et al., 2017), and 35% identity with human Dot1L (Kim et al., 2012; Figure 1A). Subsequently, the domain architectures of several Dot1 orthologs from different organisms were analyzed. The DOT1 domain, an evolutionarily conserved core structure, existed in all these orthologs. The DOT1 domain of *Po*Dot1 is 204-amino acid long and is located at the C terminus of the protein sequence like that of *S. cerevisiae* Dot1p and *A*. *flavus* Dot1, whereas human DOT1 domain is located at the N terminus of the protein sequence (Figure 1B). An AT_hook domain which is 13-amino acid long exists only in human Dot1L protein. In other important cellulolytic enzyme-producing fungi, such as *A. niger*, *T. reesei*, and *N. crassa*, we also observed putative Dot1 proteins (Figures 1A,B), but reports on their biological functions are lacking.

*Podot1* CDS was fused with *GFP* and introduced into *P. oxalicum* to replace native *Po*Dot1 (*Po*Dot1-GFP) to verify the subcellular localization of *Po*Dot1. According to the results of diagnostic PCR, the coding region and the terminator region of *Podot1* on the constructed expression cassette were homologously double-exchanged with the corresponding regions in the genome of original strain, respectively (Supplementary Image S2). This confirmed the integrity of the expression cassette and replacement of the native *Podot1* with the *Podot1-GFP*. For the subcellular localization observation of *Po*Dot1-GFP, all the *P. oxalicum* strains (including the parent strain and the PCR verified transformant *Po*Dot1-GFP) were cultivated in glucose agar media for 24 h to obtain samples for microscopy analysis. In *Po*Dot1-GFP, green fluorescence (Figure 1C, upper right) and nuclear staining (Figure 1C, bottom left) were observed. An overlap of green fluorescence and nuclear staining was observed on the merged image in the majority of hyphae (Figure 1C, bottom right, yellow arrows), thereby indicating that *Po*Dot1 is predominantly localized in the nucleus. Meanwhile, in a minority of the hyphae, GFP signal is present in the nucleus and in the cytoplasm (Figure 1D). This result was somewhat unexpected, because studies on the function of DOT1 in the cytoplasm are lacking.

### *Po*Dot1 Affects Conidiation by Regulating the Transcription of Key Regulators of Asexual Development

**FIGURE 2:**
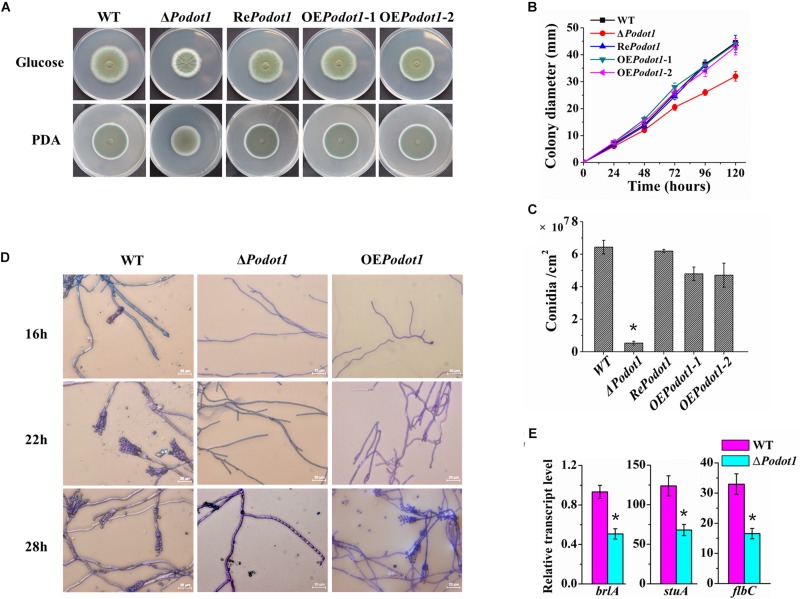
Colony morphology and conidiation of WT and *Po*Dot1-associated mutants. **(A)** Morphological characteristics of the colony in 5-day-old cultures on PDA or Vogel’s agar with 2% glucose. **(B)** Colony diameters on Vogel’s agar with 2% glucose. **(C)** Levels of conidiation of the colony in 5-day-old cultures on Vogel’s agar with 2% glucose. **(D)** Observation of the conidiophore and mycelia of WT and *Po*Dot1 mutants. **(E)** Assay of the transcription levels of *brlA*, *stuA*, and *flbC*. For each sample, three replicates were conducted. Gene expression copy numbers were calculated using the standard curves constructed for each gene, and the data were then normalized with the expression levels of the *actin* gene. Three biological triplicates were performed, and the mean values and standard deviations were calculated. Statistical analysis was performed with a one-tailed homoscedastic (equal variance) *t*-test, and *P*-values < 0.05 (^∗^) were considered statistically significant.

Classical genetic mutant strains were constructed to explore the biological effects of *Po*Dot1. Phenotypic analyses showed that *Podot1* deletion leads to asexual developmental defects. When strains were cultivated on glucose agar or PDA, the Δ*Podot1* colony wrinkled with light-green spores in the center, which was different from the dark-green colony of WT (Figure 2A). After 5 days of cultivation on glucose agar, the respective colony diameter and spore yield of the Δ*Podot1* mutant were 73.3 and 9.3% of that of WT (Figures 2B,C). The results of cover slip culture and lactophenol cotton blue staining showed the delayed initiation of conidiation due to *Po*Dot1 deletion. After 16 h of cultivation, WT started producing premature conidia. Mature conidia that have characteristic brush-like structures were formed after 22 h. For Δ*Podot1*, the initiation of asexual development did not begin until 28 h, when an abnormal structure bearing an “abacus” spore and with no brush-like structure (Figure 2D) was formed.

Three transcription factors, namely, BrlA, StuA, and FlbC, are reportedly key regulators of *P. oxalicum* asexual development. They are early regulators of conidiation, as they regulate the expression levels of pigmentation-related and spore wall protein-related genes (Qin et al., 2013a; Yao et al., 2016; Zhang et al., 2016b). They showed decreased transcription levels in Δ*Podot1* (Figure 2E), thereby suggesting that *Po*Dot1 directs conidiation by controlling their transcription. Overexpression of *Podot1* (OE*Podot1*) can produce normal brush-like structures after 28 h (Figure 2D) but displayed reduced conidiation (73.8% of WT) (Figure 2C) and delayed the initiation of conidiation. The deletion and overexpression of Dot1 and mutation of H3K79 always result in the same genetic effects, such as disrupted telomeric silencing (van Leeuwen et al., 2002; Farooq et al., 2016) and impaired cell-cycle regulation (Black et al., 2012). *Podot1* deletion and overexpression result in conidiation impairment, thereby implying that a regular amount of *Po*Dot1 protein is required for the regulation of normal asexual development.

### Expression Profiling Hints That *Po*Dot1 Regulates Mycelial Morphogenesis

A genome-wide expression profiling analysis of WT and Δ*Podot1* was performed to obtain a global view of the role of *Po*Dot1 in the regulation of gene expression. Two types of media, namely, glucose and cellulose, were used to cultivate the strains. The glucose medium provides a basic culture condition for mycelial growth and repressed cellulase formation, and the cellulose medium is ideal for the induction of cellulolytic enzyme gene expression. Saturation analysis, principal component analysis (PCA), and Pearson correlations of each sample indicated their availability for omics analysis (Supplementary Images S3–S5).

**FIGURE 3:**
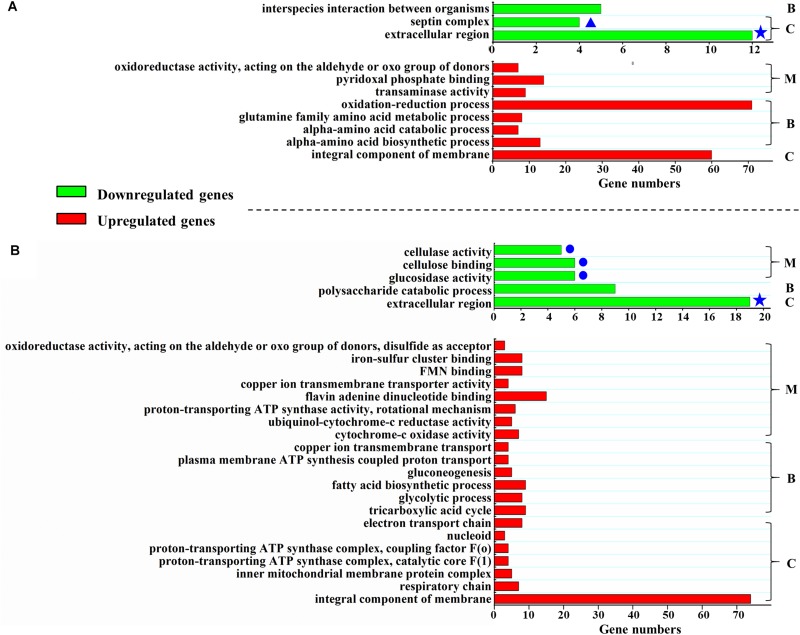
Function enrichment of differentially expressed genes in Δ*Podot1*. Function enrichment of the upregulated or downregulated (more than or equal to twofold, probability ≥ 0.8) genes in Δ*Podot1* compared with that of WT after the strains were cultivated in the glucose medium **(A)** or in the cellulose medium **(B)**. B, C, and M indicate the GO category. B, biological process; C, cellular component; M, molecular function. Green bars, function enrichment analysis of downregulated genes. Red bars, function enrichment analysis of the upregulated genes. Blue stars indicate the same category of enrichment in both media. Blue dots indicate the clustering of three “molecular function” GO entries that are all associated with polysaccharide degradation. Significantly different expression between samples were identified through a significance test with combined thresholds (diverge probability ≥ 0.8, fold change ≥ 2) (Audic and Claverie, 1997). GO annotation and function enrichment analysis were performed by GO database (http://www.geneontology.org/) and Blast2GO with threshold at FDR ≤ 0.05 (Conesa et al., 2005).

When strains are cultivated in glucose medium, the expression levels of 800 genes in Δ*Podot1* and WT significantly differed (fold change ≥ 2, probability ≥ 0.8) (Supplementary Table S2). Among the regulated genes, 319 genes (39.9%) were downregulated in Δ*Podot1* compared with those in the WT. GO analysis revealed that the downregulated genes in Δ*Podot1* were involved mainly in the septin complex, extracellular region (GO category: cellular component), and interspecies interaction between organisms (GO category: biological process) (Figure 3A, green bars). A total of 481 genes (60.1%) were upregulated in Δ*Podot1* compared with those in the WT (Figure 3A, red bars).

**FIGURE 4:**
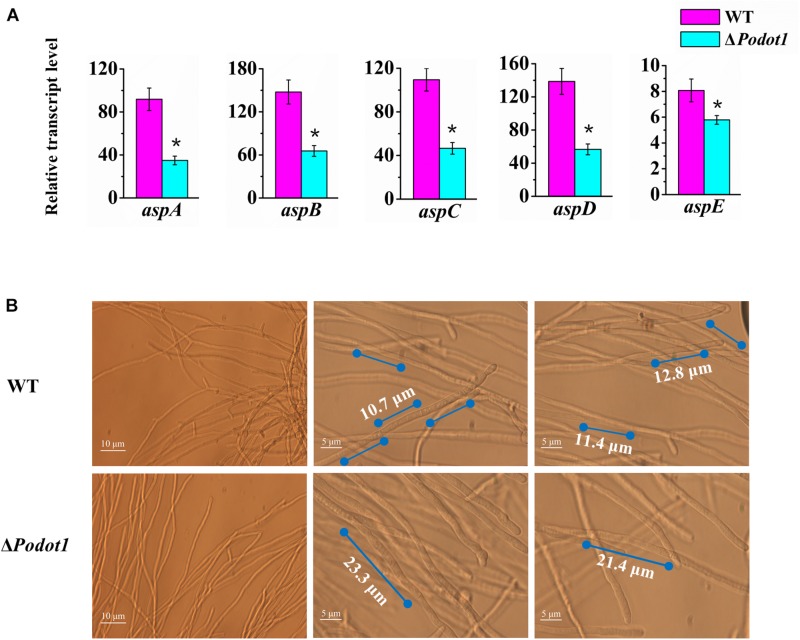
Analysis of septa and branch formation in wild-type strains and Δ*Podot1* mutants. **(A)** Analysis of the transcription level of five septin encoding genes. Statistical analysis was performed with a one-tailed homoscedastic (equal variance) *t*-test, and *P*-values < 0.05 (^∗^) were considered statistically significant. **(B)** Observation of mycelial branches and interval of septa.

Based on expression profiling, the genes involved in septin complex were enriched downregulated in Δ*Podot1* when cultivated in the glucose medium (Figure 3A, blue triangle). In the *P. oxalicum* genome, five septin-encoding genes, namely, *aspA* (GenBank EPS34854.1, an ortholog of *S. cerevisiae* CDC11), *aspB* (GenBank EPS34223.1, an ortholog of *S. cerevisiae* CDC3), *aspC* (GenBank EPS34492.1, an ortholog of *S. cerevisiae* CDC12), *aspD* (GenBank EPS31868.1, an ortholog of *S. cerevisiae* CDC10), and *aspE* (GenBank EPS26861.1, found only in filamentous fungi), were present and showed consistent downregulated transcription in Δ*Podot1* (Figure 4A). Does the downregulation of septin-encoding genes result in impairment of septum formation?

Septa form at regular intervals along the lengths of fungi hyphae. If present, they can be observed under a light microscope. A dramatic decrease was observed in their emergence in the Δ*Podot1* mutant compared to that in the WT. The distance between septa in *P. oxalicum* WT was 11.63 ± 1.67 μm, which was almost similar to that (12.84 ± 1.9 μm) in the *Aspergillus* group (Gajjar et al., 2013). The distance between septa was larger in Δ*Podot1* (21.87 ± 1.27 μm), which was almost twice that in WT (Figure 4B). We also observed that the number of branches in the Δ*Podot1* mutant was lower compared with that in the WT. The *L*_*hgu*_ of Δ*Podot1* mutant was >180.0 μm/tip, which was higher than that of the WT (99.0 ± 1.27 μm/tip). *Po*Dot1 affects mycelial morphology by interfering with septum and branch formation.

### Expression Profiling Suggests That *Po*Dot1 Extensively Regulates Glycoside Hydrolase Gene Expression

When strains are cultivated in cellulose medium, the expression levels of 787 genes in Δ*Podot1* and WT were significantly different (fold change ≥ 2, probability ≥ 0.8) (Supplementary Table S3). Among the regulated genes, 448 (56.9%) and 339 (43.1%) genes were upregulated and downregulated, respectively, in Δ*Podot1* compared with those in the WT. GO analysis revealed that the downregulated genes in Δ*Podot1* are mainly involved in cellulase activity, cellulose binding, glucosidase activity (GO category: molecular function) (Figure 3B, blue dots), and polysaccharide catabolic process (GO category: biological process). The extracellular region was also enriched (GO category: cellular component) (Figure 3B, green bars). The downregulated genes (GO category: molecular function) and their predicted functions are listed in Supplementary Table S4.

**FIGURE 5:**
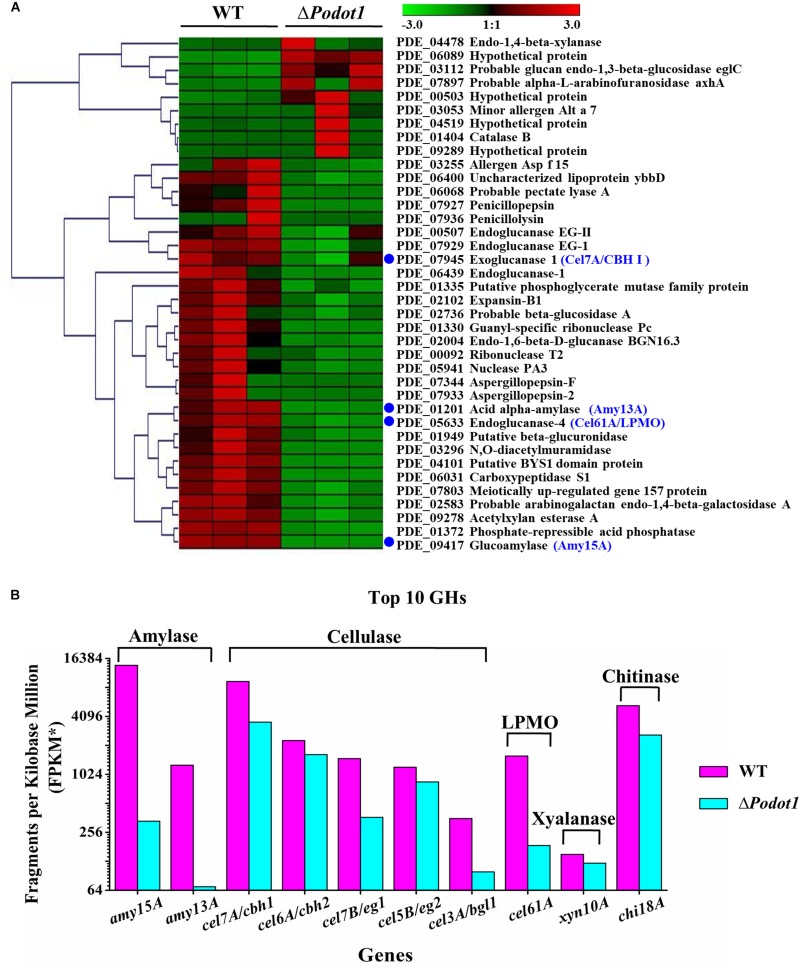
Comparative analysis of expression profiles of the secreted proteins in WT and Δ*Podot1*. **(A)** Clustering analysis of genes encoding the secreted proteins using Genesis (Sturn et al., 2002). The gradient color bar code at the top indicates a value at log2 fold change of expression in the treatment case to the expression in the control case. Blue dots, the top 4 extracellular glycoside hydrolases assayed in *P. oxalicum* secretome. **(B)** Expression levels of the top 10 extracellular glycoside hydrolases encoding genes. The copy number of unambiguous transcripts for each gene was normalized to FPKM.

The “extracellular region” was enriched in cellulose and glucose media (Figure 3, green bars with blue stars), but the glucose medium is not good for extracellular protein synthesis. Did the deletion of *Po*Dot1 affect extracellular proteins, especially extracellular cellulases? We noticed that among the 110 secreted proteins determined by *P. oxalicum* secretome analysis (Liu et al., 2013), 39 secreted protein-encoding genes were differentially expressed (fold change ≥ 2, probability ≥ 0.8). Among the 39 secreted proteins, 30 were downregulated (Figure 5A) including glucoamylase Amy15A (PDE_09417, GenBank No. EPS34453.1), α-amylase Amy13A (PDE_01201, GenBank No. EPS26265.1), cellobiohydrolase Cel7A/CBHI (PDE_07945, GenBank No. EPS32984.1), and lytic polysaccharide monooxygenases (LPMO) Cel61A (PDE_05633, GenBank No. EPS30681.1) (Figure 5A, blue dot). They were the top four extracellular GHs in *P. oxalicum* secretome, and their products accounted for >50% of the total extracellular protein of *P. oxalicum* (Liu et al., 2013).

What about the other important extracellular proteins? In addition to the aforementioned top 4 extracellular GHs, the expressions of the other six extracellular GHs that belong to the top 10 secretion amounts in *P*. *oxalicum* secretome (Liu et al., 2013) were analyzed. These included chitinase Chi18A (GenBank No. EPS33160.1, PDE_08122), β-glucosidase Cel3A/BGLI (GenBank No. EPS27792.1, PDE_02736), xylanase Xyn10A (GenBank No. EPS33132.1, PDE_08094), endoglucanase Cel7B/EGI (GenBank No. EPS32968.1, PDE_07929), endoglucanase Cel5B/EGII (GenBank No. EPS34262.1,PDE_09226), and cellobiohydrolyase Cel6A/CBHII (GenBank No. EPS32164.1, PDE_07124) (Figure 5B). The transcriptome data (FPKM) showed that 10 genes, including two amylase genes (*amy15A* and *amy13A*), five cellulase genes (*cel7A*/*cbh1*, *cel6A*/*cbh2*, *cel7B*/*eg1*, *cel5B*/*eg2*, and *cel3A*/*bgl1*), a cellulose-active LPMO gene (*cel61A*), a xylanase gene (*xyl10A*), and a chitinase gene (*chi18A*), were downregulated (Figure 5B) in Δ*Podot1*, and their expression levels were 2.4, 5.5, 37.7, 71.4, 24.5, 70.4, 27.9, 11.7, 81.0, and 49.5%, respectively, compared with those in the WT. The deletion of *Podot1* led to downregulated transcription of prominent extracellular GH-encoding genes in *P. oxalicum*.

### The Deletion of *Podot1* Results in Decreased Synthesis of Extracellular GHs

Glycoside hydrolase production is tightly controlled at the transcriptional level (Lynd et al., 2008). Thus, we questioned whether the downregulated transcription level of prominent extracellular GH-encoding genes (Figure 5) results in decreased synthesis of extracellular GHs. Therefore, the activities of multiple GHs in the WT- and *Po*Dot1-related mutants (Δ*Podot1*, Re*Podot1*, and OE*Podot1*) were detected in agar (solid) and fermentation (liquid) media.

**FIGURE 6:**
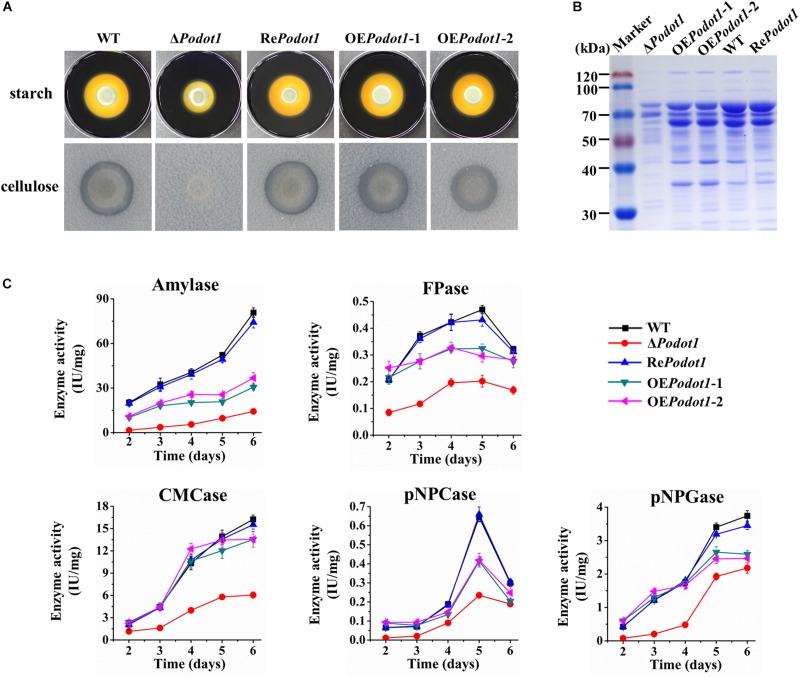
Assay of multiple glycoside hydrolase activities of WT and *Po*Dot1-associated mutants (Δ*Podot1*, Re*Podot1*, and OE*Podot1*) in solid and liquid media. **(A)** Observation of amylolytic and cellulolytic halo around the colonies. **(B)** SDS-PAGE analysis of extracellular secreted proteins in liquid fermentation medium supplemented with bran and microcrystalline cellulose. **(C)** Amylase activities and cellulase activities in the liquid fermentation medium. Enzyme activities were normalized to the ratio of corresponding biomass (IU/mg). One enzyme activity unit was defined as the amount of enzyme required for producing 1 μmol glucose or pNP per minute under the assayed conditions. Three biological triplicates were performed for all enzyme analysis. The mean values and standard deviations were calculated. Statistical analysis was performed, and *P*-values < 0.05 were considered statistically significant.

When strains are cultivated on Vogel’s agar supplemented with 2% starch or 0.5% cellulose, the Δ*Podot1* colony was observed with diminished amylolytic and cellulolytic halo compared with WT (Figure 6A), implying less amylase or cellulolytic enzyme secretion in Δ*Podot1*. When strains are cultivated in liquid fermentation medium added with wheat bran and cellulose, which were ideal for GH production, the fermentation supernatant of Δ*Podot1* had significantly fainter protein bands, especially in the range of 35–116 kDa, than that of WT (Figure 6B). This range was an area for GH aggregation, where prominent amylases and cellulases (cellobiohydrolases, endoglucanases, and β-glucosidases) were located according to previous reports (Liu et al., 2013). The OE*Podot1* mutant showed almost identical extracellular protein profiling as the WT, thereby suggesting that the native expression level of *Podot1* was sufficient for normal GH production.

The levels of amylase, filter paper (FPA, indicating overall cellulase activity), CMCase (indicating endoglucanase activity), pNPCase (indicating cellobiohydrolyase activity), and pNPGase (indicating extracellular β-glucosidase activity) activities and extracellular protein concentration were assayed. Enzyme activities were normalized by intracellular protein concentration (indicating the biomass level) (Figure 6C) to exclude the influence of biomass differences of different strains. The absence of *Po*Dot1 resulted in significantly decreased amylase and cellulase activities compared with WT. On the 5th day, the amylase, FPA, CMCase, pNPCase, and pNPGase activities of Δ*Podot1* were 18.5, 43.0, 41.6, 36.4, and 56.6%, respectively, of those of WT. Consistent with the decrease in the abovementioned GH activities, the extracellular protein concentration of Δ*Podot1* decreased to 58.2% of that of WT. *Podot1* overexpression strains (OE*Podot1*-1 and OE*Podot1*-2) showed almost identical or even lower levels of amylase and cellulase activities compared with WT (Figure 6C). The absence of *Po*Dot1 remarkably repressed the synthesis of extracellular GHs.

### Downregulated GH Production in Δ*Podot1* Was Accompanied by Defects of H3K79 Methylation

**FIGURE 7:**
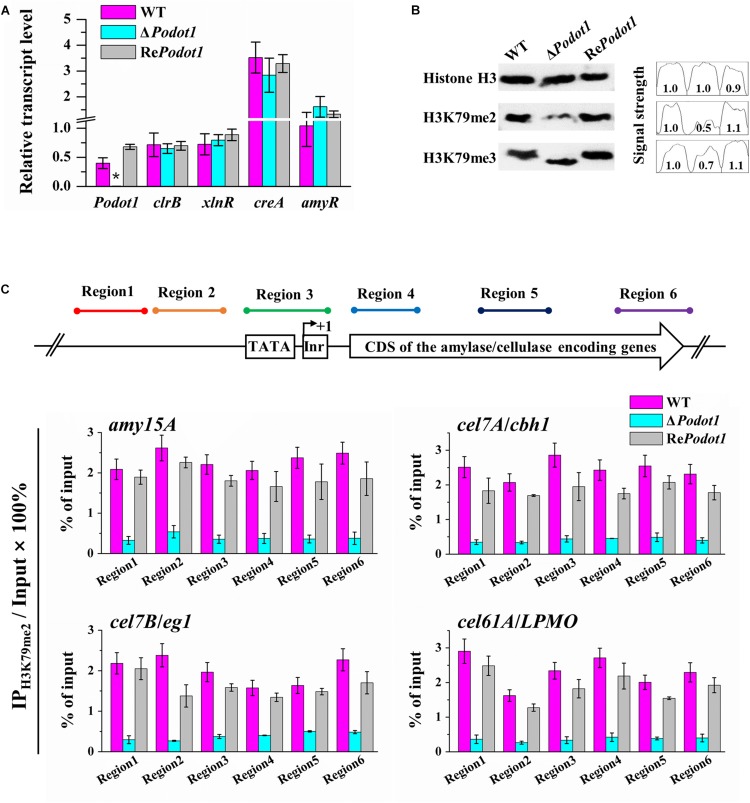
Analysis transcription level of TF encoding genes and H3K79 methylation in *Po*Dot1 mutants. **(A)** Expression level assay of genes encoding four TFs (*clr-2*/*clrB*, *xyr1*/*xlnR*, *amyR*, and *cre1*/*creA*) via qRT-PCR analysis. *actin* gene was used for data normalization. **(B)** Assay of the global histone methylation patterns at H3K79 in WT and Δ*Podot1* through Western blot. Histone H3 was used as the loading control. **(C)** Analysis of the methylation modification level of H3K79me2 on the specific regions of four target genes (*amy15A*, *cel7A*/*cbh1*, *cel7B*/*eg1*, and *cel61A*/*LPMO*) through ChIP-qPCR. Equal amounts of extracted chromatin (1 mg/IP) of each sample were used for IP reactions, and 0.1 mg chromatin DNA was used as input (without IP) for each sample. The purified IP products and input DNA were subjected to quantitative PCR (qPCR). For each gene, the transcription start site (TSS) was designated as +1. Six typical regions (Region 1–Region 6) were focused: Region 1 and Region 2 were orderly located at ∼500 bp upstream (−) of the TSS. Region 3 covered the initiator and the TATA box. Region 4, Region 5, and Region 6 were orderly located in the downstream (+) of TSS, that is, in the 5′-region of the coding domain sequences (CDS), in the middle of the CDS, and in the 3′-region of the CDS, respectively. The relative enrichment of IP DNA was calculated by % of input. The values showed the means of the three biological replicates, and the error bar indicated standard deviation.

The foregoing results led us to explore the mechanism of how *Po*Dot1 regulates GH gene expression. Several conserved positive or negative TFs involved in GH gene expression, such as positive regulators encoded by *xyr1*/*xlnR* and *clr-2*/*clrB* (Mach-Aigner et al., 2008; Coradetti et al., 2012) and negative regulators encoded by *cre1*/*creA* and *amyR* (Li et al., 2015), have been well characterized. The deletion or overexpression of these TFs can regulate GH gene expression remarkably. Therefore, we speculated that *Po*Dot1 affects GH production by regulating the expression of key TFs. However, no significant difference (*P-*value > 0.05) was observed in the expression levels of these TFs after detection by qRT-PCR (Figure 7A), thereby suggesting that the repressed synthesis of extracellular GHs caused by *Po*Dot1 deletion was not mediated by these TFs.

As a putative histone methyltransferase, *Po*Dot1 is predicted to methylate histone H3 lysine 79 (H3K79). In yeast, majority of H3 is methylated mainly in H3K79me2 and H3K79me3 states (Schubeler et al., 2004; Frederiks et al., 2008). Therefore, we detected the global levels of H3K79 (H3K79me2 and H3K79me3) methylation levels in the WT and *Po*Dot1 mutants by Western blotting (Figure 7B). A remarkable reduction in H3K79me2 level (to 50% signal strength of WT) and a slight reduction in H3K79me3 level (to 70% signal strength of WT) were observed in Δ*Podot1*.

Western blot analysis results represented the global levels of histone methylation modification in chromatin. Local analysis of H3K79 methylation at specific GH gene loci was subsequently performed by ChIP-qPCR. Based on transcriptome and enzyme activity measurements, four representative genes, including one amylase-encoding gene *amy15A*, two cellulase-encoding genes *cel7A*/*cbh1* and *cel7B*/*eg1*, and one cellulase-related LPMO gene *cel61A*/*LPMO*, showed significantly downregulated transcription levels and were selected as targets for histone methylation level analysis at individual gene loci. For each gene, six typical regions (Regions 1–6) that cover upstream sequences and CDS were the focus. Regions 1 and 2 were orderly located at approximately 500 bp upstream (−) of the transcription start site (TSS). Region 3 covered the initiator and TATA box. Regions 4–6 were orderly located in the downstream (+) of TSS, that is, in the 5′-region, in the middle, and in the 3′-region of CDS (Figure 7C), respectively. ChIP-qPCR results obviously showed that H3K79me2 in promoter (Regions 1–3) and CDS (Regions 4–6) regions of the selected four gene loci were impaired due to *Po*Dot1 deletion. Specifically, the H3K79me2 levels at the 5′-region of CDS (region 4) of *amy15A*, *cel7A*/*cbh1*, *cel7B*/*eg1*, and *cel61A*/*LPMO* were reduced to only 18.1, 18.7, 25.6, and 15.6%, respectively, in Δ*Podot1* compared with those in the WT (Figure 7C). Therefore, the defects of H3K79 methylation may be the key reason of the downregulated transcription level of GH-encoding genes in Δ*Podot1*.

### Results of TAP–MS Hint That *Po*Dot1 Has No Direct Interaction With Transcription Machinery but Interacts With COMPASS

Previous results showed that *Po*Dot1 is involved in the transcriptional regulation of genes. Does *Po*Dot1 play a regulatory role by interacting with transcription machines? TAP–MS was conducted in *Po*Dot1-labeled strain (*Po*Dot1-HA-FLAG) to identify the protein–protein interaction collaborator of *Po*Dot1.

In addition to *Po*Dot1 itself, 14 proteins (unique pep count ≥ 3) with existing putative physical interactions with *Po*Dot1 were captured (Table 1 and Supplementary Image S6 and Supplementary Table S5): (1) two proteins associated with methylation, including *S*-adenosylmethionine (SAM) synthetase ETH-1 (PDE_07230) that is responsible for the synthesis of methyl donors SAM, and Swd2, one of the subunits of complex associated with Set1 (COMPASS); (2) five proteins associated with translation, including translation elongation factor EF-2 subunit (PDE_08649), tyrosyl-tRNA synthetase (PDE_00455), and three ribosomal proteins (60S ribosomal proteins L5, L22, and L24); (3) four proteins associated with energy transfer and redox reactions, including F-type H^+^-transporting ATPase subunit alpha, quinone oxidoreductase Pig3, glutamate dehydrogenase GdhA, and pyruvate decarboxylase PdcA; and (4) three heat shock proteins and chaperones, including chaperonin GroEL (Hsp60), multifunctional chaperone (14-3-3 family), and molecular chaperone HtpG (Hsp90).

**TABLE 1:** Putative proteins interacting with *Po*Dot1 identified through TAP-MS experiments (unique pep count ≥ 3).

**Gene ID (locus_tag)**	**Annotation in *P. oxalicum***	**Homolog in *S. cerevisiae***	**Identities (%)**	**Subcellular localization^∗^**
**Methylation associated**				
PDE_07451	Histone methyltransferase Swd2; WD repeat protein	SWD2	33	Nucleus
PDE_07230	*S*-Adenosylmethionine synthetase; ETH-1	SAM2	76	Cytoplasm
**Translation associated**				
PDE_08649	Translation elongation factor EF-2 subunit	EFT2	78	Cytoplasm
PDE_00455	Tyrosyl-tRNA synthetase	TYS1	47	Nucleus/cytoplasm
PDE_00374	60S ribosomal protein L5	RPL5	63	Nucleus/cytoplasm
PDE_04825	60S ribosomal protein L22	RPL22A	50	Nucleus/cytoplasm
PDE_03397	60S ribosomal protein L24	RPL24B	74	Nucleus/cytoplasm
**Energy transfer and REDOX associated**				
PDE_06476	F-type H+-transporting ATPase subunit alpha	ATP1	79	Mitochondrion
PDE_02985	NADPH2: quinone reductase	ZTA1	30	Nucleus/cytoplasm
PDE_06117	Glutamate dehydrogenase GdhA	GDH1	66	Nucleus
PDE_04873	Pyruvate decarboxylase PdcA	PDC1	47	Nucleus/cytoplasm
**Heat shock proteins and chaperones**				
PDE_04315	Chaperonin GroEL; heat shock protein Hsp60	HSP60	72	Cytoplasm/mitochondrion
PDE_05280	Multifunctional chaperone (14-3-3 family)	BMH2	82	Nucleus/cytoplasm
PDE_09149	Molecular chaperone HtpG; heat shock protein 90	HSC82	83	Cytoplasm/mitochondrion/plasma membrane

*^∗^Subcellular localization information obtained from the Saccharomyces Genome Database (SGD) (https://www.yeastgenome.org).*

**FIGURE 8:**
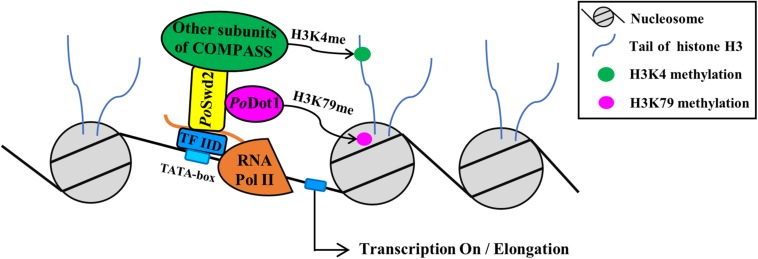
The schema of *Po*Dot1 indirectly interacting with transcription machinery via the mediation of COMPASS. As shown by TAP results, *Po*Dot1 has no direct interaction with transcription machinery. However, it interacts with *Po*Swd2, one of the subunits of COMPASS [contain subunits of Swd1, Swd2, Swd3, Bre2, Sdc1, Spp1, Sgh1, and Set1 (Miller et al., 2001)]. It is worth noting that the subunits of RNA Pol II and general TF TFIID can be captured when *Po*Swd2 is used as bait to identify protein–protein interaction (Li et al., 2019). Therefore, *Po*Dot1 indirectly interacts with transcription machinery, and this interaction was mediated by COMPASS.

Among the 14 proteins, RNA Pol II subunit or general TF was not found, thereby suggesting that *Po*Dot1 does not directly interact with transcription machinery. An evidence that yDot1 directly interacts with transcription machinery in yeast was also absent. However, *Po*Swd2, one of the subunits of complex associated with Set1 (COMPASS), which contain at least eight subunits (i.e., Swd1, Swd2, Swd3, Bre2, Sdc1, Spp1, Sgh1, and Set1) (Miller et al., 2001), was found in TAP–MS results, thereby suggesting the interaction of *Po*Dot1 with COMPASS (Figure 8). Interestingly, among them, we observed cytoplasm- or mitochondrion-localized proteins, such as SAM synthetase and translation elongation factor EF-2 subunit (Table 1). This result was unexpected because of the lack of studies on the function of Dot1 in the cytoplasm thus far. However, considering previously observed dual subcellular localization of *Po*Dot1 in the cytoplasm and nucleus (Figures 1C,D), *Po*Dot1 may have some unexpected functions in the cytoplasm.

## Discussion

In filamentous fungi, the production of GH is tightly regulated by the level of transcription. Transcription factors play an important role in the transcriptional regulation of GH-encoding genes (Li et al., 2015). In addition, more and more evidence indicates that chromatin regulation is involved in the activation or inhibition of GH gene transcription (Mello-de-Sousa et al., 2015, 2016; Cao et al., 2019; Li et al., 2019). In *T. reesei*, there is a change in nucleosome localization near the CAE (cbh2 activating element) motif upstream of the cellulase gene *cbh2*; mutation of the cellulase gene transcriptional regulator Cre1 makes increased accessibility of DNA in the core promoter region of cellulase gene *cbh1* (Mello-de-Sousa et al., 2015); in addition, multiple genes involved in chromatin remodeling and histone modification were found to be significantly upregulated in Cre1 mutants under cellulose-induced conditions (Hakkinen et al., 2014). These findings suggest that chromatin modifications are closely related to the transcriptional regulation of cellulase genes. Although the histone lysine methylation is an important part of histone modification that is widely associated with a range of key cellular processes (Kouzarides, 2002), its function in the regulation of cellulolytic enzyme genes is poorly elucidated. A putative methyltransferase Lae1 is a positive regulator of the transcription of cellulolytic genes in *T. reesei* (Seiboth et al., 2012), but its histone target and regulation mechanism are yet to be determined (Patananan et al., 2013). It remains to be clarified that whether (and how) various types of histone methylation modification are involved in the transcriptional regulation of cellulose-degrading fungi.

As histone modifiers, Dot1 and H3K79 have been previously reported to be associated with active transcription (Steger et al., 2008), but their involvement in transcription is not fully understood. We originally speculated that the extensive repression of extracellular GH-encoding genes caused by *Po*Dot1 deletion was mediated by some key TFs, such as positive regulators XlnR and ClrB and negative regulators CreA and AmyR. However, our results showed that gene transcription downregulation was not related to these TFs (Figure 7A). In contrast, we found H3K79 methylation is indispensable for the active transcription of GH-encoding genes. We observed that H3K79me2 in all six regions of the four gene loci, e.g., at and around the TSS (Regions 1–3) to CDS (Regions 4–6) regions, were impaired due to the absence of *Po*Dot1 (Figure 7C), thereby indicating that the repressed GH genes in Δ*Podot1* may be affected in the simultaneous process of transcriptional initiation and elongation. This result is slightly different from that in yeast, as yeast Dot1 and H3K79 methylations were mainly detectable at the coding regions of active genes (Shahbazian et al., 2005). As the inactivation of H3K79 methylation is always associated with specific chromatin structures and is important for heterochromatin formation and maintenance of the silencing regions of chromatin in yeast (Osborne et al., 2009; Takahashi et al., 2011), it was assumed that a decrease in H3K79 methylation level, specifically a remarkable reduction in H3K79me2 in Δ*Podot1*, resulted in changes in chromatin structure and affected transcription.

As shown by TAP results, *Po*Dot1 has no direct interaction with transcription machinery. However, it interacts with *Po*Swd2, one of the subunits of COMPASS. Yeast Dot1 was also reported to have a direct physical interaction with Swd2 using Affinity Capture-Western (Lee et al., 2007). It is worth noting that the subunits of RNA Pol II (i.e., Pol II subunits A, B, C, and E) and general TF TFIID can be captured when *Po*Swd2 is used as bait to identify protein–protein interaction (Li et al., 2019). Therefore, *Po*Dot1 indirectly interacts with transcription machinery, and this interaction was mediated by COMPASS (Figure 8). Interestingly, the methylation modification on H3K4 (specifically H3K4me2 and H3K4me3) by COMPASS subunit Set1 was recently verified as an essential active marker for GH synthesis (Li et al., 2019). However, a significant change on H3K4 methylation in Δ*Podot1* was not detected (data not shown), thereby indicating that the absence of *Po*Dot1 did not affect the function of COMPASS.

According to the expression profiling, the genes involved in septin complex were enriched down regulated in Δ*Podot1* when cultivated in the glucose medium. Filamentous ascomycetes, such as *A. nidulans* and *N. crassa*, form multinucleated hyphae that are compartmentalized by cross-walls and septa (Steinberg et al., 2017). During septa formation, septins, a conserved family of GTP-binding proteins, function as morphogenetic scaffolds that recruit and organize other proteins at sites of septum formation and polarized growth (Mouriño-Pérez and Riquelme, 2013). Septins AspA, AspB, and AspC in *A. nidulans* are localized as rings and collars at septa and branches, and are required for normal septation and organized development (Lindsey et al., 2010; Hernández-Rodríguez et al., 2012). In the *P. oxalicum*, the downregulation of septin-encoding genes resulted in impairment of septum formation in Δ*Podot1*. Although the functions of septum formation and mycelium compartmentalization are still up for debate, some researchers surmise that septation can launch polar growth by branching, forming one branch per compartment (Dynesen and Nielsen, 2003). For example, the disruption of AspB results in delayed and defective branching in *A. nidulans* (Hernández-Rodríguez et al., 2012). In the *P. oxalicum*, the downregulation of five septin-encoding genes also resulted in a decrease in the number of mycelial branches in Δ*Podot1*. The results indicated that *Po*Dot1 interfered with septum and branch formation by regulating the expression of septin-encoding genes, thereby affecting mycelial morphology.

In the majority of hyphae, *Po*Dot1 was observed to be predominantly localized in the nucleus (Figure 1C), which was expected, because Dot1 is a nuclear protein associated with chromosomes and is distributed throughout the chromatin (Ontoso et al., 2014). Meanwhile, in a minority of the hyphae, GFP signal is present in the nucleus and in the cytoplasm (Figure 1D). Among the enriched proteins interacting with *Po*Dot1, cytoplasm-localized proteins were observed (Table 1), which was somewhat unexpected, because the function of Dot1 in the cytoplasm has not been reported thus far.

In *S. cerevisiae*, more than 20 proteins reportedly have physical interaction with Dot1, and most of them are nuclear proteins (Altaf et al., 2007; Oh et al., 2010; Lee et al., 2018). However, some existing proteins that are primarily located in the cytoplasm, such as coronin (CRN1), protein involved in cytoplasm vesicular transport (IMH1) (Krogan et al., 2006), and palmitoylated plasma membrane-bound casein kinase I (YCK1) (Ptacek et al., 2005), have physical interaction with Dot1. In addition, some recently characterized lysine (K)-specific MTases (methyltransferases) (KMTs) belonging to the same seven-β-strand (7BS) MTases as DOT1 (Falnes et al., 2016) perform methylation in the cytoplasm, thereby methylating lysine in non-histone proteins (Demirci et al., 2008; Owings et al., 2016). Most 7BS KMTs identified in *S. cerevisiae*, such as elongation factor methyltransferase (Efm) 2 and 3, reportedly target translation elongation factors eEF2 (Davydova et al., 2014) and Efm4-7 and translation elongation factors eEF1A (Couttas et al., 2012; Jakobsson et al., 2015). It is worth noting that the translation elongation factor EF-2 subunit (PDE_08649) was observed in the TAP results of *Po*Dot1. Aside from its main roles in the nucleus, *Po*Dot1 may have functions similar to other 7BS KMTs, i.e., performing methylation in the cytoplasm. More detailed and in-depth studies should be conducted to fully understand *Po*Dot1 functions.

## Data Availability Statement

The datasets generated for this study can be found in the DDBJ/EMBL/GenBank under the accession number AGIH00000000, and NCBI’s Gene Expression Omnibus (GEO) database under the accession number GSE136585.

## Author Contributions

YQin conceived and designed the study. YL conducted the experiments. YH and KZ were involved in data acquisition. YP, YQu, and JZ were involved in data analysis. YQin and YL wrote the manuscript. All authors revised and approved the manuscript.

## Conflict of Interest

The authors declare that the research was conducted in the absence of any commercial or financial relationships that could be construed as a potential conflict of interest.

## Footnotes

1 https://www.ncbi.nlm.nih.gov/

2 http://smart.embl-heidelberg.de/

3 http://pfam.xfam.org/

4 http://www.geneontology.org/

5 https://www.blast2go.com/

## Supplementary Material

IMAGE S1 Strategy and results of PCR and Southern blot analysis for the verification of multiple mutants. **(A)** Strategy and results of PCR for the verification of *Podot1* deletion strain. **(B)** Strategy and results of PCR for the verification of *Podot1* overexpression strains. **(C)** Strategy and results of Southern blot analysis for the verification of *Podot1* deletion strain. **(D)** Strategy and results of Southern blot analysis for the verification of *Podot1* overexpression strain.

IMAGE S2 Strategy**(A)** and results **(B)** of diagnostic PCR for the verification of *Po*Dot1-GFP strain.

IMAGE S3 Saturation analysis of the depth of transcriptome sequencing data. **(A)** Three biological replicates of WT cultivated in a glucose medium. **(B)** Three biological replicates of Δ*Podot1* cultivated in a glucose medium. **(C)** Three biological replicates of WT cultivated in cellulose medium. **(D)** Three biological replicates of Δ*Podot1* cultivated in cellulose medium.

IMAGE S4 Principle component analysis (PCA) results of the samples of RNA-seq. Each dot represents one sample on the principal component value, which are named as Component 1 (the first component), Component 2 (the second component), Component 3 (the third component), and Component 4 (the fourth component). **(A)** Component 1 virus Component 2. **(B)** Component 3 virus Component 4. Samples can be clustered according to the value distribution of two components.

IMAGE S5 Heatmap of Pearson correlation coefficient values acrossing samples of RNA-seq. The correlation value between each two samples was calculated based on normalized expression result. Gradient color bar code at the right indicates the minimum value in white and the maximum in blue. If one sample is highly similar with another one, the correlation value between them is very close to 1.

IMAGE S6 Verification of *Po*Dot1 extracted in TAP-MS experiments via silver staining and Western blot analysis. **(A)** Silver staining of TAP-tagged proteins together with associated proteins after affinity purification. **(B)** Western blot analysis of affinity-purified tagged *Po*Dot1.

TABLE S1 Primers used in this study.

TABLE S2 800 genes exhibited significant differences (fold change ≥ 2, probability ≥ 0.8) between Δ*Podot1* and WT after cultivation in glucose medium.

TABLE S3 787 genes exhibited significant differences (fold change ≥ 2, probability ≥ 0.8) between Δ*Podot1* and WT after cultivation in cellulose medium.

TABLE S4 List of downregulated genes (≥ 2-fold, FDR < 0.05) in Δ*Podot1* compared with WT with significantly enriched GO terms (GO category: molecular function) when cultivated for 24 h on the condition of cellulose medium.

TABLE S5 Mass spectrometry identification of proteins obtained by TAP-MS experiments of *Po*Dot1.
