# Supplementary figures and images for: The Indispensable Role of Histone Methyltransferase PoDot1 in Extracellular Glycoside Hydrolase Biosynthesis of Penicillium oxalicum
Source: Front Microbiol. 2019 Nov 7;10:2566. doi: 10.3389/fmicb.2019.02566 (PMC6853848; doi:10.3389/fmicb.2019.02566)

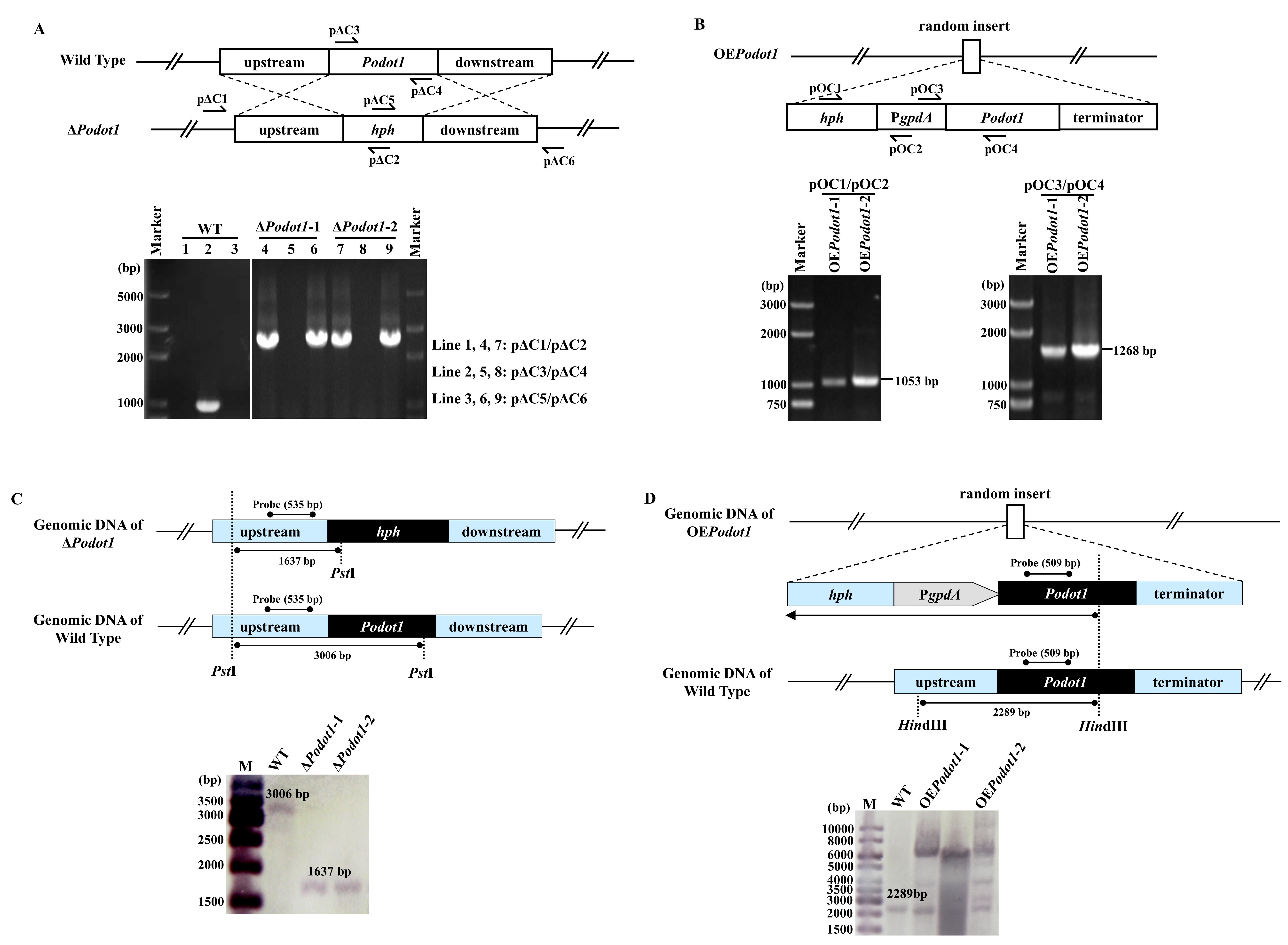

Supplement: IMAGE S1 — Strategy and results of PCR and Southern blot analysis for the verification of multiple mutants. (A) Strategy and results of PCR for the verification of Podot1 deletion strain. (B) Strategy and results of PCR for the verification of Podot1 overexpression strains. (C) Strategy and results of Southern blot analysis for the verification of Podot1 deletion strain. (D) Strategy and results of Southern blot analysis for the verification of Podot1 overexpression strain. [file Image_1.TIF]

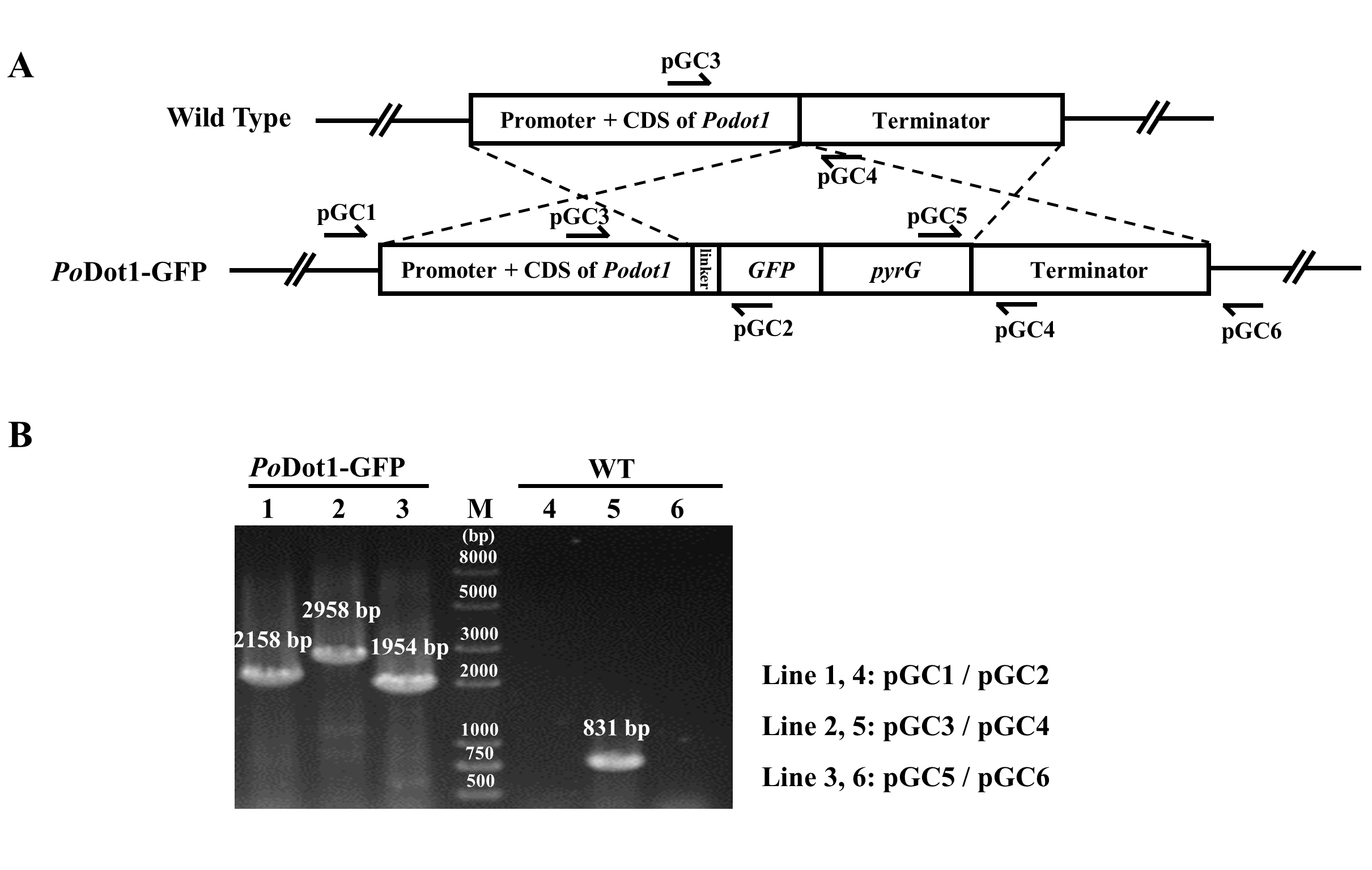

Supplement: IMAGE S2 — Strategy(A) and results (B) of diagnostic PCR for the verification of PoDot1-GFP strain. [file Image_2.TIF]

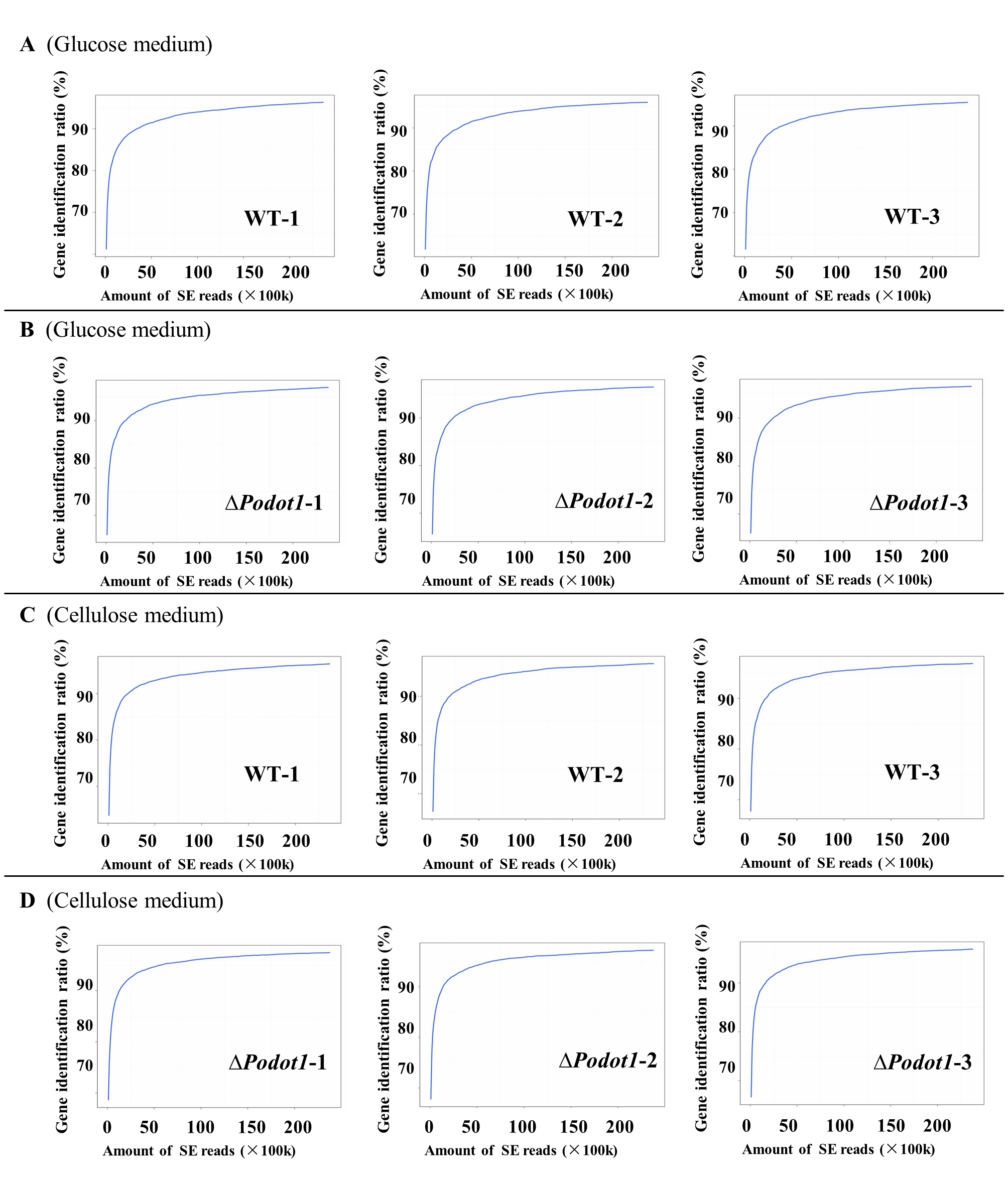

Supplement: IMAGE S3 — Saturation analysis of the depth of transcriptome sequencing data. (A) Three biological replicates of WT cultivated in a glucose medium. (B) Three biological replicates of ΔPodot1 cultivated in a glucose medium. (C) Three biological replicates of WT cultivated in cellulose medium. (D) Three biological replicates of ΔPodot1 cultivated in cellulose medium. [file Image_3.TIF]

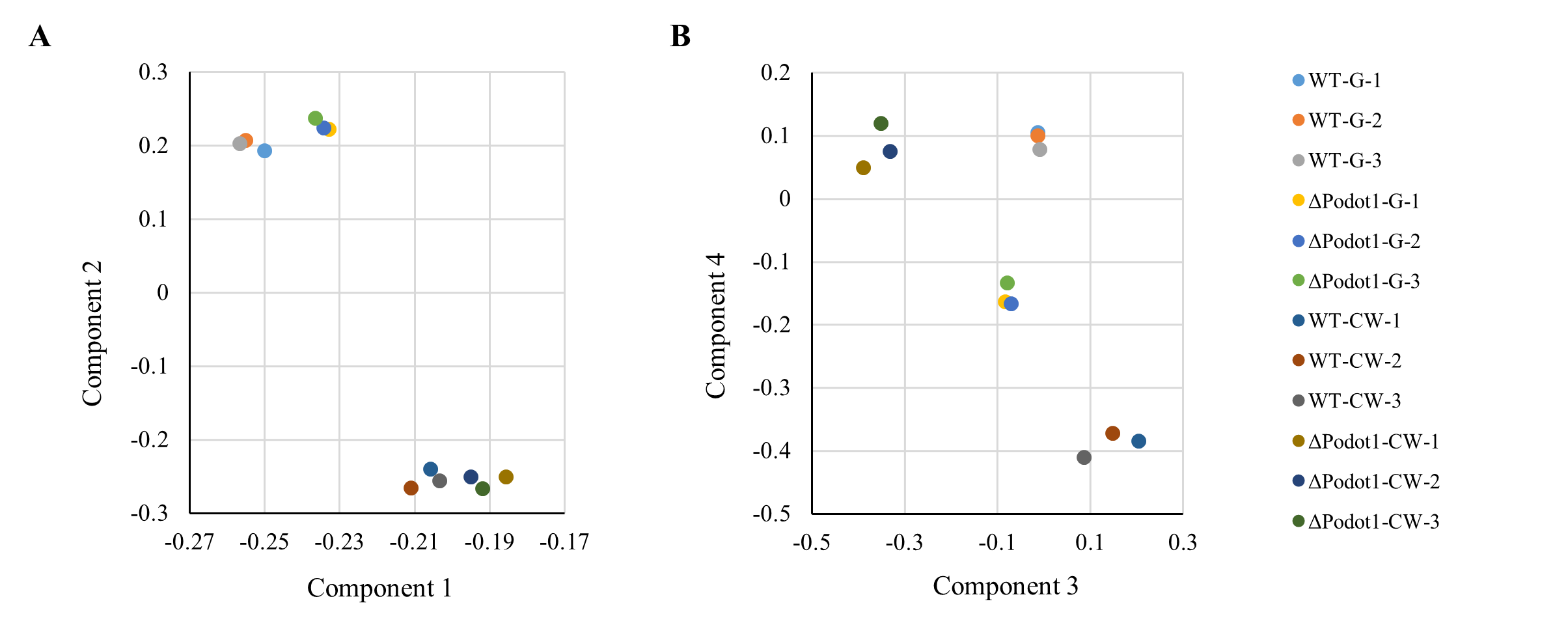

Supplement: IMAGE S4 — Principle component analysis (PCA) results of the samples of RNA-seq. Each dot represents one sample on the principal component value, which are named as Component 1 (the first component), Component 2 (the second component), Component 3 (the third component), and Component 4 (the fourth component). (A) Component 1 virus Component 2. (B) Component 3 virus Component 4. Samples can be clustered according to the value distribution of two components. [file Image_4.TIF]

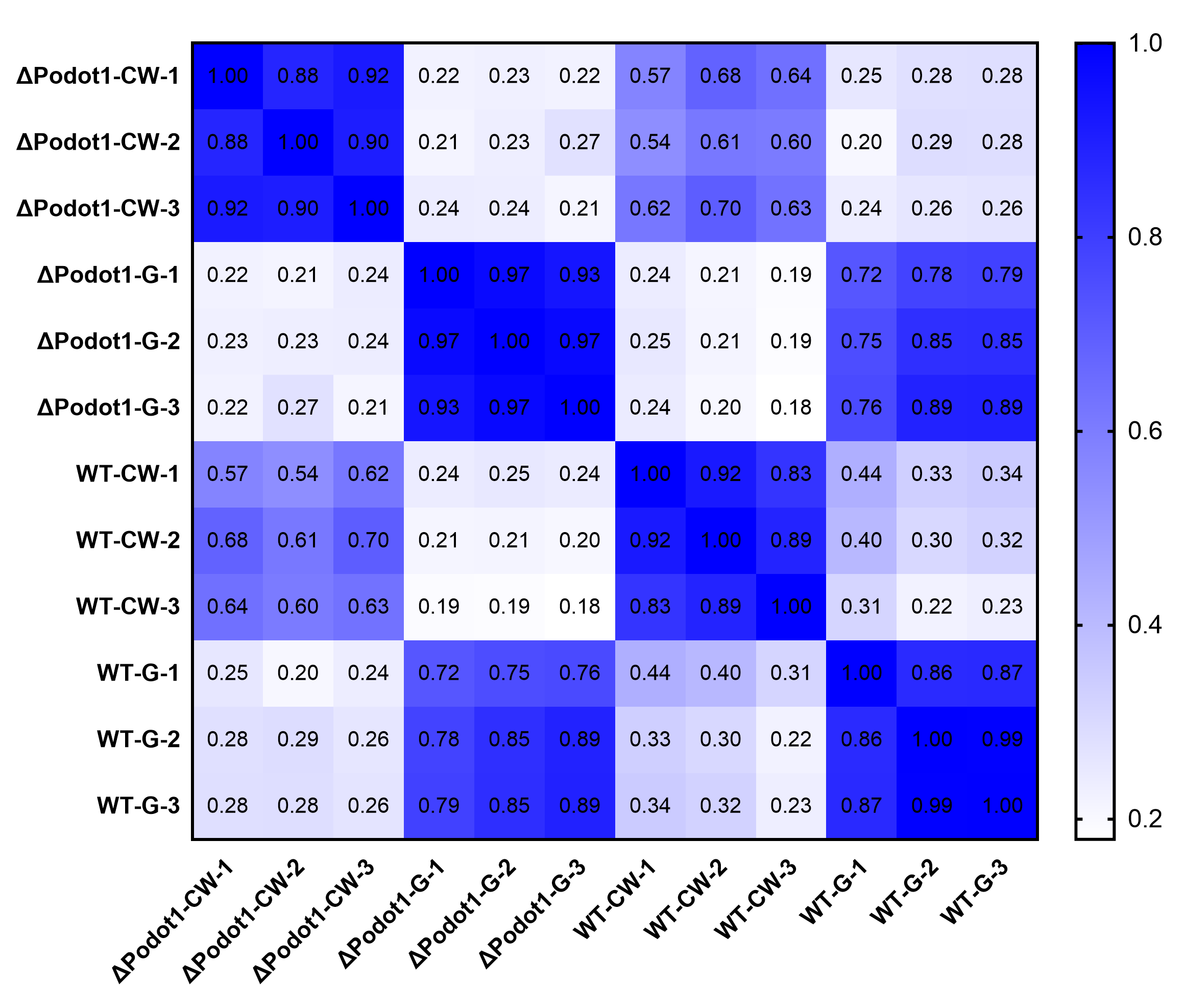

Supplement: IMAGE S5 — Heatmap of Pearson correlation coefficient values acrossing samples of RNA-seq. The correlation value between each two samples was calculated based on normalized expression result. Gradient color bar code at the right indicates the minimum value in white and the maximum in blue. If one sample is highly similar with another one, the correlation value between them is very close to 1. [file Image_5.TIF]

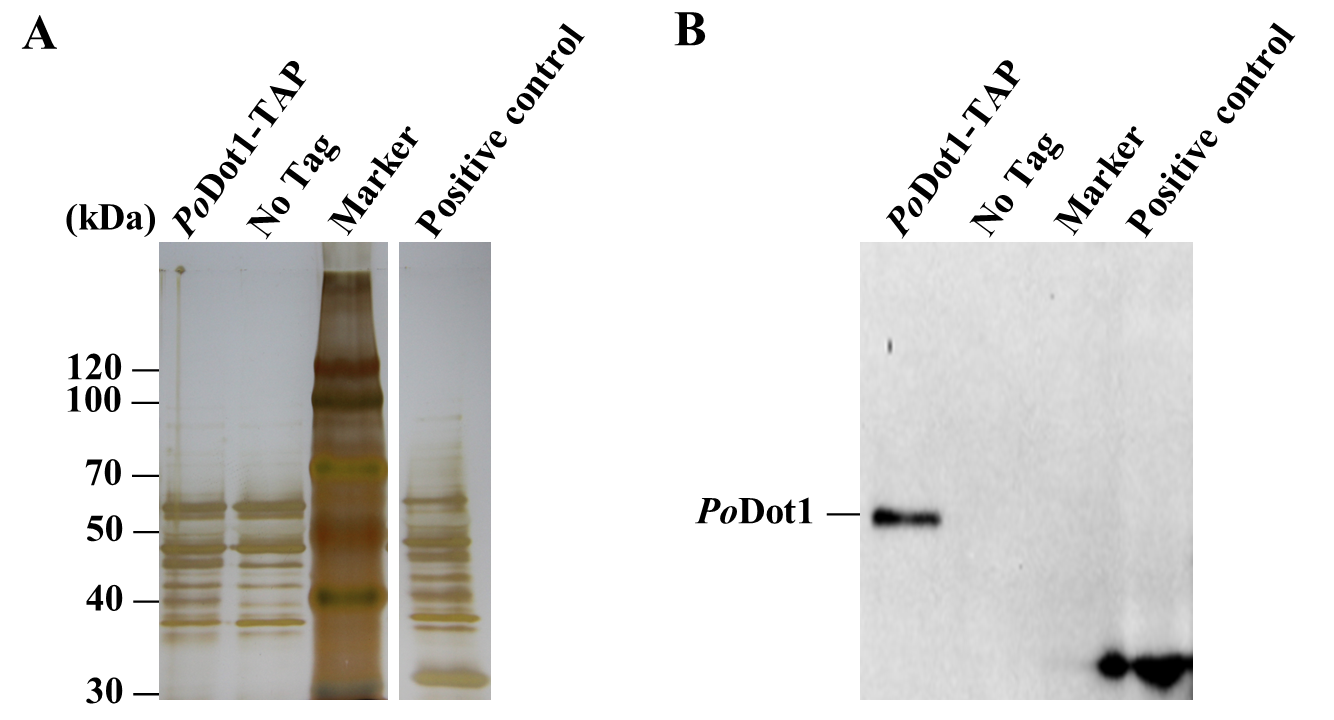

Supplement: IMAGE S6 — Verification of PoDot1 extracted in TAP-MS experiments via silver staining and Western blot analysis. (A) Silver staining of TAP-tagged proteins together with associated proteins after affinity purification. (B) Western blot analysis of affinity-purified tagged PoDot1. [file Image_6.TIF]
