# Supplementary material for: The Indispensable Role of Histone Methyltransferase PoDot1 in Extracellular Glycoside Hydrolase Biosynthesis of Penicillium oxalicum
Source: Front Microbiol. 2019 Nov 7;10:2566. doi: 10.3389/fmicb.2019.02566 (PMC6853848; doi:10.3389/fmicb.2019.02566)
Supplement: TABLE S1 — Primers used in this study. [file Table_1.DOCX]

**Supplementary Table 1. Primers used in this study**

| **Primer name** | **Sequence (5'-3')** |
| --- | --- |
| **Primers for the construction of Δ*Podot1*** | |
| pΔdot1-UF | GATGGATATCGGCATGGAACAGATC |
| pΔdot1-UR | CTCCTTCAATATCAGTTAACGTCGGGAGATGCACAAATATAAACAGCAG |
| pΔdot1-DF | CCGTCACCAGCCCTGGGTTGGCTAGACTGTCGAGAGTGTTGGTCG |
| pΔdot1-DR | GAACGCACATATTGGTTCATCAAGC |
| phph-F | CGACGTTAACTGATATTGAAGGAG |
| phph-R | CAACCCAGGGCTGGTGACGG |
| PΔdot1-NF | CACCCTCATCAGTCAAATCCAAGTC |
| pΔdot1-NR | GTGCTGTCTAGGTCAACACAGAGAAC |
| **Primers for the construction of Re*Podot1*** | |
| ptrA-F | GGGCAATTGATTACGGGATC |
| ptrA-R | ATGGGGTGACGATGAGCCGC |
| pRedot1-F | GCGGCTCATCGTCACCCCATGATGGGTTCTCTGAAGACAACAAAAG |
| pRedot1-R | GTCTCTCATCTGTCTCTGAAACTGAC |
| pRedot1-NF | GGGCAATTGATTACGGGATC |
| pRedot1-NR | CCATTTGACAATAGACACTCGCTTG |
| **Primers for the construction of OE*Podot1*** | |
| pgpdA-F | CCGTCACCAGCCCTGGGTTGAGTCAGACGGCGTAACCAAAAGTC |
| pgpdA-R | GGTGATGTCTGCTCAAGCGG |
| pOEdot1-F | CCGCTTGAGCAGACATCACCATGGGGTTTTTTGACCACCTCC |
| pOEdot1-R | CAAGCTACACACCACTTGCGAAACG |
| phph-F | CGACGTTAACTGATATTGAAGGAG |
| phph-R | CAACCCAGGGCTGGTGACGG |
| pOEdot1-NF | CGACGTTAACTGATATTGAAGGAG |
| pOEdot1-NR | CCAACCTCGCGTTCTACGAGGCTTG |
| **Primers for the construction of *Po*Dot1-GFP** | |
| pGdot1-UF | GCGCTGCTTATCGATTACCCTTTTGC |
| pGdot1-UR | CAGCTCCTCGCCCTTGCTCACCATTCCTCCTCCTCCCGTAGAGTCATCGATAAAAGCTTTC |
| pGFP-F | ATGGTGAGCAAGGGCGAGGAGCTG |
| pGFP-R | TTACTTGTACAGCTCGTCCATGCCG |
| pyrG-F | CGGCATGGACGAGCTGTACAAGTAAGCAACTTCCTCGAGAACGCG |
| pyrG-R | CCCTTTTAGTCAATACCGTTAC |
| pGdot1-DF | GTAACGGTATTGACTAAAAGGGGTGGCGAGGATTTTCACATATGCAG |
| pGdot1-DR | GTGCTGTCTAGGTCAACACAGAGAAC |
| pGdot1-NF | CGCTCTTTATTCATATACAGGCGCTG |
| pGdot1-NR | GGATCCACTGATGCACGAAGGTTTCG |
| pGC1 | CACAATAATCCTTCGTCTCATTGATC |
| pGC2 | GCTCGATGCGGTTCACCAGGGTGTCG |
| pGC3 | GTTGATTAACAATCAAGCTTTCACTC |
| pGC4 | GAAAGTTGGACTGTCACGTAGTCCAC |
| pGC5 | GTACACGGCACGCTCGGTTGAGTACG |
| pGC6 | CAGTTAATCGGGAGATCTCAAGTAAG |
| **Primers for the amplification of probes of Southern blot** | |
| SP-F1 | GAATTTGAAAGATACCCGAAGACCG |
| SP-R1 | GGAGATGCACAAATATAAACAGCAG |
| SP-F2 | GAGGAGCGTTCTCCTTGCAGGCG |
| SP-R2 | CTTCCTGGAGGCGCTGGGATACTG |
| **Primers for RT-qPCR** | |
| Actin-QF | GTTCCATTCTCGCCTCCCTCT |
| Actin-QR | AGAAGCACTTGCGGTGAACGA |
| Dot1-QF | GCGAAAAGCATCAAAAAACGG |
| Dot1-QR | TTTGACTCTGTCGCCGAAGCC |
| BrlA-QF | GTCAGGAACATCTCAAGCGGC |
| BrlA-QR | GCTTGCTGTGGGTCTTGGTG |
| CreA-QF | TGGGTACGAGTGAACTCCATCTT |
| CreA-QR | TGTGACCTTGACCAGGACTGTAA |
| ClrB-QF | AGCACAAGTCGAGATGGGATT |
| ClrB-QR | CGCTTGCTGGCTTCGTAAAT |
| XlnR-QF | CGATCCGCTCTTGCCCAGGTA |
| XlnR-QR | GGGCGAGAACTTCACGTCTG |
| AmyR-QF | TCACCATCGGCAACTTTCTCC |
| AmyR-QR | TCGCCTTCCATGTGAATCTCG |
| **Primers for ChIP-qPCR** | |
| Amy15A-1F | GCCAGGGCGTGCGAGGAGTCTTG |
| Amy15A-1R | CATCTGAGCACTTCTTCAGATTC |
| Amy15A-2F | CAGATAATCCTGAGATGCAACGG |
| Amy15A-2R | GCATTAGCGTCTTGACCGTGAAG |
| Amy15A-3F | GGTCAGTGTCATGCAATGTTGCG |
| Amy15A-3R | CGAAACTTGATCAGTGAACTGAG |
| Amy15A-4F | GTCTCGACTTCTCTACGCACTGG |
| Amy15A-4R | CTTGGAGTAGGCACCGCTGGAGC |
| Amy15A-5F | CTCCTCAGATTCTCTGCTACATG |
| Amy15A-5R | CTGGAAAGTGACGTCGTCGCACG |
| Amy15A-6F | CTCTGTGGTACGCCACGGTCAAC |
| Amy15A-6R | GGTGTCGTTCTCGGTAGCAGTAG |
| Cbh1-1F | CATATCCTTCACCGAGTGGGTTG |
| Cbh1-1R | GACGATGGAGGCGAAATTGAGG |
| Cbh1-2F | CTGGGGAGCTTCCTCCGTGTCCG |
| Cbh1-2R | CAATTCGCCTCTCGACTGATTTC |
| Cbh1-3F | CTTCAGGATCCACCCAAGGAAG |
| Cbh1-3R | GTGATGGATTGGATCAAAGATC |
| Cbh1-4F | GTTCCATCTCCTACCAGATCTAC |
| Cbh1-4R | GTGCAGCTGCCACCGGCAGAGCAC |
| Cbh1-5F | CTCACTCCTCACCCTTGCGACAC |
| Cbh1-5R | GTAGAAGTTGGTCACACCCTGAC |
| Cbh1-6F | CCACTGCTGCCTCTGGCGGTAAC |
| Cbh1-6R | CACTGGGAGTAGTACTCGTTCTG |
| Eg1-1F | CAGATGCATCATCTCTCACCTG |
| Eg1-1R | CCTTGGGGTGAAACTCTTCAAC |
| Eg1-2F | GCGTCTCCGGATCGTTTCGCTATG |
| Eg1-2R | GAAGGAACACGGAGACTTGACCAG |
| Eg1-3F | GTCGAAATACGACAGAAATGATG |
| Eg1-3R | GTTGAGCAAACTTGGCAATGAGC |
| Eg1-4F | CTTTCACAAGGAGACAGTTCCTC |
| Eg1-4R | CCGACGTGTCCTGAGCAACACAG |
| Eg1-5F | GTACGGTTCAGGATACTGCGACG |
| Eg1-5R | GTCATCGGAGCTGCAAGGGTGAG |
| Eg1-6F | GCTCTGGATCGAGCTCGTCTACC |
| Eg1-6R | GCGCCTTGCAGGTAGTTCCGGAC |
| LPMO-1F | CAGCCACGCTGGTTGAATCCCTG |
| LPMO-1R | GTATGAAATTCAGACGCTTGCTAG |
| LPMO-2F | GTGGTAGAGAAATATCCGACGTC |
| LPMO-2R | GACAGGGATAAATCCAAGATTC |
| LPMO-3F | CATTACCGGTTATGATTCCAGTG |
| LPMO-3R | GGTTTGAATAGATTCGGCCTGAG |
| LPMO-4F | CCACCACCCTATCTGCCATGTTG |
| LPMO-4R | GAGATAGCCAGTGTAGCTGGACG |
| LPMO-5F | CCGCAGGCTCCAAGGTGGAAATC |
| LPMO-5R | CAATCAGACCGGCCTCATCGATC |
| LPMO-6F | CAGTGTTTGAATCTGCAGGTCAC |
| LPMO-6R | CACGCGCTGTACAGAGCTGGACC |
