# Supplementary material for: The Indispensable Role of Histone Methyltransferase PoDot1 in Extracellular Glycoside Hydrolase Biosynthesis of Penicillium oxalicum
Source: Front Microbiol. 2019 Nov 7;10:2566. doi: 10.3389/fmicb.2019.02566 (PMC6853848; doi:10.3389/fmicb.2019.02566)
Supplement: TABLE S4 — List of downregulated genes (≥ 2-fold, FDR < 0.05) in ΔPodot1 compared with WT with significantly enriched GO terms (GO category: molecular function) when cultivated for 24 h on the condition of cellulose medium. [file Table_4.DOC]

**Supplementary Table 4. List of downregulated genes (≥ 2-fold, FDR < 0.05) in Δ*Podot1* compared with WT with significantly enriched GO terms (GO category: molecular function) when cultivated for 24 h on the condition of cellulose medium.**

| **GO-ID** | **Term** | **Gene ID**  **(locus_tag)** | **Description of putative *P. oxalicum* ORF** |
| --- | --- | --- | --- |
| GO:0008810 | Cellulase activity | PDE_00507 | Endoglucanase EG-II |
|  |  | PDE_05633 | Endoglucanase-4 |
|  |  | PDE_06439 | Endoglucanase-1 |
|  |  | PDE_07928 | Endoglucanase-5 |
|  |  | PDE_07929 | Endoglucanase EG-1 |
| GO:0015926 | Glucosidase activity | PDE_01756 | Alpha-glucosidase |
|  |  | PDE_02004 | Endo-1,6-beta-D-glucanase |
|  |  | PDE_02583 | Probable arabinogalactan endo-1,4-beta-galact -osidase A |
|  |  | PDE_02736 | Probable beta-glucosidase A |
|  |  | PDE_04151 | Alpha-glucosidase |
|  |  | PDE_09417 | Glucoamylase |
| GO:0030248 | Cellulose binding | PDE_00507 | Endoglucanase EG-II |
|  |  | PDE_02102 | Expansin-B1 |
|  |  | PDE_07928 | Endoglucanase-5 |
|  |  | PDE_07929 | Endoglucanase EG-1 |
|  |  | PDE_07945 | Exoglucanase 1 |
|  |  | PDE_09278 | Acetylxylan esterase A |
